# Immunostimulatory RNA leads to functional reprogramming of myeloid-derived suppressor cells in pancreatic cancer

**DOI:** 10.1186/s40425-019-0778-7

**Published:** 2019-11-06

**Authors:** Philipp Metzger, Sabrina V. Kirchleitner, Michael Kluge, Lars M. Koenig, Christine Hörth, Carlotta A. Rambuscheck, Daniel Böhmer, Julia Ahlfeld, Sebastian Kobold, Caroline C. Friedel, Stefan Endres, Max Schnurr, Peter Duewell

**Affiliations:** 1 Center of Integrated Protein Science Munich (CIPSM) and Division of Clinical Pharmacology, Klinikum der Universität München, Lindwurmstrasse 2a, 80337 Munich, Germany; 2 Department of Neurosurgery, University Hospital, LMU Munich, 81377 Munich, Germany; 3 Institute for Informatics, Ludwig-Maximilians-Universität München, 80333 Munich, Germany; 4 Institute of Innate Immunity, University of Bonn, Venusberg-Campus 1, 53127 Bonn, Germany

**Keywords:** MDSC, Type I IFN, Poly(I:C), MDA5, Pancreatic cancer, Immunotherapy

## Abstract

**Background:**

The tumor microenvironment (TME) combines features of regulatory cytokines and immune cell populations to evade the recognition by the immune system. Myeloid-derived suppressor cells (MDSC) comprise populations of immature myeloid cells in tumor-bearing hosts with a highly immunosuppressive capacity. We could previously identify RIG-I-like helicases (RLH) as targets for the immunotherapy of pancreatic cancer inducing immunogenic tumor cell death and type I interferons (IFN) as key mediators linking innate with adaptive immunity.

**Methods:**

Mice with orthotopically implanted Kras^G12D^ p53^fl/R172H^ Ptf1a-Cre (KPC) pancreatic tumors were treated intravenously with the RLH ligand polyinosinic-polycytidylic acid (poly(I:C)), and the immune cell environment in tumor and spleen was characterized. A comprehensive analysis of the suppressive capacity as well as the whole transcriptomic profile of isolated MDSC subsets was performed. Antigen presentation capability of MDSC from mice with ovalbumin (OVA)-expressing tumors was investigated in T cell proliferation assays. The role of IFN in MDSC function was investigated in *Ifnar1*^*−/−*^ mice.

**Results:**

MDSC were strongly induced in orthotopic KPC-derived pancreatic cancer, and frequencies of MDSC subsets correlated with tumor weight and G-CSF serum levels, whereas other immune cell populations decreased. Administration of the RLH-ligand induced a IFN-driven immune response, with increased activation of T cells and dendritic cells (DC), and a reduced suppressive capacity of both polymorphonuclear (PMN)-MDSC and monocytic (M)-MDSC fractions. Whole transcriptomic analysis confirmed an IFN-driven gene signature of MDSC, a switch from a M2/G2- towards a M1/G1-polarized phenotype, and the induction of genes involved in the antigen presentation machinery. Nevertheless, MDSC failed to present tumor antigen to T cells. Interestingly, we found MDSC with reduced suppressive function in *Ifnar1*-deficient hosts; however, there was a common flaw in immune cell activation, which was reflected by defective immune cell activation and tumor control.

**Conclusions:**

We provide evidence that the treatment with immunostimulatory RNA reprograms the TME of pancreatic cancer by reducing the suppressive activity of MDSC, polarizing myeloid cells into a M1-like state and recruiting DC. We postulate that tumor cell-targeting combination strategies may benefit from RLH-based TME remodeling. In addition, we provide novel insights into the dual role of IFN signaling in MDSC’s suppressive function and provide evidence that host-intrinsic IFN signaling may be critical for MDSC to gain suppressive function during tumor development.

## Background

Pancreatic ductal adenocarcinoma (PDAC) is predicted the second most frequent cause for cancer-associated death in the Western world [1]. However, no immunotherapeutic approach has been approved for PDAC so far [2]. A hallmark of tumors is the immunosuppressive network with the recruitment of immune cell populations that effectively dampen T cell function and promote tumor growth. Thus, there is a great unmet need towards a better understanding of the suppressive tumor microenvironment (TME) and its role for immunotherapeutic failure.

Chronic low-grade inflammation is a known risk factor for carcinogenesis and is involved in desmoplastic conversions characterized by a high infiltration of non-malignant stromal and immune cells [3, 4]. The frequency of peripheral blood myeloid cells correlates with the disease stage of PDAC patients [5] and the infiltration of macrophages, neutrophils and regulatory T cells (T_reg_) into the tumor serves as a negative prognostic marker for survival [6]. Based on immune cell infiltration, tumors can be classified as “cold” tumors, in which immune cell infiltration - especially T cells - is sparse; those tumors mostly fail to respond to immunotherapies [7, 8]. On the other hand, “hot” tumors are densely infiltrated with T cells, which indicates an immunological active TME susceptible for immunotherapy with checkpoint inhibitors. PDAC creates an immune-privileged TME that is characterized by low T cell frequencies that lack functionality to fight cancer cells due to a negative immune regulation in the TME [9, 10]. Along this classification, PDAC is a graving example of a “cold” tumor [11].

An interesting approach for turning “cold” tumors “hot” could be reprogramming of the TME into an immune-permissive state. PDAC show high frequencies of *Kras* mutations with high secretion of growth factors such as granulocyte-macrophage colony-stimulating factor (GM-CSF) and granulocyte colony-stimulating factor (G-CSF), which are responsible for emergency myelopoiesis recruiting myeloid cells into the TME [12, 13]. Myeloid cells, such as monocytes and granulocytes, are pathologically activated by tumor-intrinsic inflammatory signals and acquire T cell suppressive functions [14]. This pathological activation led to the introduction of the functional classification of MDSC into monocytic (M)-MDSC and polymorphonuclear (PMN)-MDSC [15].

MDSC promote tumor growth and metastasis via various mechanisms including PD-L1-dependent direct inhibition of T cell function and amino acid deprivation by arginase-1 and iNOS [15–17]. Macrophages can either be polarized into a pro-inflammatory anti-microbial M1 state or into an anti-inflammatory tissue remodeling M2 state depending on the stimulus [18]. Based on that, similar mechanisms have been proposed for tumor-associated neutrophils (TAN), placing TGF-β as an inducer of tumor-promoting N2 neutrophils [19] and IFN-β as an inducer of anti-tumor N1 neutrophils [20]. Efforts to specifically target MDSC mostly focused on preventing recruitment and function by blocking stem cell or colony-stimulating factors, arginase-1 or the iNOS pathway [21]. Thus, switching myeloid cells from a suppressive into an immune-supporting phenotype might serve as an option for restoring anti-tumor immunity. The FDA-approved vitamin A derivate all-trans retinoic acid (ATRA) has been shown to stimulate myeloid cell maturation into functionally active and T cell-promoting cells, thus, reprogramming the suppressive MDSC phenotype [22]. Another approach is the induction of type I IFN signaling in tumor hosts, which has been demonstrated to reduce the suppressive capacity of myeloid cells [23, 24].

IFN plays a central role in the immunogenicity of tumor cell death and it also seems to directly affect MDSC function [23, 25]. We could previously show that RIG-I-like helicases (RLH) induce a potent IFN-driven immune response with the induction of immunogenic tumor cell death. Stimulation with synthetic RLH ligands led to enhanced cross-presentation of tumor antigen by dendritic cells (DC) and a robust expansion of cytotoxic T cells [26, 27]. RLH ligands have emerged as promising candidates for tumor immunotherapy and have entered phase I/Ib clinical trials for the treatment of advanced solid tumors (NCT03739138, NCT02828098). Moreover, modifications of the RIG-I ligands, combining siRNA-targeted gene silencing with RIG-I activation, have already been evaluated in preclinical models and show enhanced tumor control [28–30].

Here, we aim at characterizing the role of MDSC during RLH-based immunotherapy, using the MDA5/RLH ligand polyinosinic-polycytidylic acid poly(I:C), complexed to PEI (poly(I:C)_c_) for intracellular delivery, in an orthotopic model of pancreatic cancer. Whole transcriptomic analysis of MDSC populations revealed an IFN pathway-enriched gene signature, accompanied by a shift from a M2/G2- towards a M1/G1-polarized phenotype. Using IFN receptor 1 (IFNAR1)-deficient mice, we show that IFNAR signaling may play an important role during MDSC development in tumor-bearing hosts, promoting a suppressive phenotype. Our data provide evidence that re-programming of MDSC via RLH-based immunotherapy contributes to unleashing T cell-mediated tumor control.

## Material and methods

### Mice

Female C57BL/6 mice were obtained from Janvier (France). All mice were kept with a 12-h light/dark cycle, water ad lib. and regular chow diet (sniff, Soest, Germany), at the University of Munich, Munich, Germany. The Kras^G12D^ p53^fl/R172H^ Ptf1a-Cre (KPC)-derived T110299 pancreatic tumor cell line was provided by Prof. Jens Siveke, (University Hospital Essen, Germany), *Ifnar1*^−/−^ mice (*Ifnar1*^*tm1Agt*^) were provided by Prof. Simon Rothenfußer (LMU Munich, Germany). Age- and sex-matched 6–12 weeks old wild type mice and OT-I TCR-transgenic mice (*C57BL/6*^*Tg(TcraTcrb)1100Mjb/J*^) were purchased from Jackson Laboratory (Stock number 003831).

### Cell culture

Primary cells were cultured in RPMI-1640 medium (Sigma-Aldrich, Taufkirchen, Germany), supplemented with 10% fetal calf serum (FCS), 2 mM L-glutamine, 100 U/l penicillin, 0.1 mg/ml streptomycin, 100 mM non-essential amino acids (all gibco®, Thermo Fisher Scientific, Karlsruhe, Germany), 1 mM sodium pyruvate and 50 mM 2-mercaptoethanol (both Sigma Aldrich). Tumor cells were cultured in DMEM high glucose media (Sigma-Aldrich), supplemented with 10% FCS, 2 mM L-glutamine, 100 U/l penicillin and 0.1 mg/ml streptomycin. OVA expression T110299 cell were generated by transfection with the pAc-Neo-OVA plasmid [31] using the Novagen Genejuice® transfection reagent, according to the manufacturer’s instructions. OVA+ T110299 cells were selected with G418 (geneticin). All cells were kept in a humidified incubator at 37 °C and 5% CO_2_. For the assessment of MHC-I expression of explanted tumors, we made use of EpCAM-expressing T110299 tumors cells, which were generated by transducing T110299 cell with a pMXs vector harboring murine EpCAM, thus, allowing labeling with mAb for flow cytometry analysis.

### Orthotopic tumor induction and poly(I:C)_c_ treatment

Orthotopic tumors were induced by surgical implantation, as described before [28]. Briefly, mice were anesthetized and, by surgical incision, the pancreas was carefully mobilized for injection. After the injection of 2 × 10^5^ T110299 cells in 25 μl PBS, the pancreas was relocated and the incision was closed by surgical suture. Mice were monitored daily and distressed mice were sacrificed. For treatment, 50 μg VacciGrade™ HMW polyinosinic-polycytidylic acid (poly(I:C)) (InvivoGen, Toulouse, France) were complexed with in vivo-jetPEI® (VWR International GmbH, Darmstadt, Germany), at a N/P ratio of 6 in 5% glucose solution, according to the manufacturer’s instructions (referred to as poly(I:C)_c_). Mice were treated i.v. at day 18 and 20 after tumor induction with either poly(I:C)_c_ or glucose as control. 6 h after the first treatment cytokine levels of CXCL10 and IL-6 were measured using enzyme-linked immunosorbent assays (ELISA) from R&D systems (Minneapolis, USA). 12 h after the second treatment IFNβ levels were measured using ELISA from R&D systems. On day 14 and day 21 following tumor induction, serum G-CSF levels were measured by ELISA (R&D systems, Bio-Techne GmbH, Wiesbaden-Nordenstadt, Germany). Tumor-free mice served as controls. All other serum cytokines were measured 6 h after the first treatment by multiplex analysis, using a Procarta Plex Mix&Match Panel (Invitrogen, Thermo Fisher Scientific, Karlsruhe, Germany) and a MAGPIX® system (Merck, Darmstadt, Germany), according to the manufacturer’s protocol.

### Cell isolation

Spleens were processed through a 70 μm cell strainer, followed by red blood cell lysis (BD Pharm Lyse™, BD Biosciences, Heidelberg, Germany). Tumor tissue was minced into pieces and mechanically dissociated using the mouse Tumor Dissociation Kit with the gentleMACS™ Dissociator application (both Miltenyi Biotech, Bergisch Gladbach, Germany), according to the manufacturer’s instructions. The cell suspension was separated from tissue debris by sequentially using 100 μm and 70 μm cell strainers. For functional assays, T cells were isolated using the Pan T cell isolation Kit II and stained with 2.5 μM CFSE (Thermo Fisher Scientific, Karlsruhe, Germany) for 4 min at room temperature. For MDSC isolation, the Myeloid-Derived Suppressor Cell Kit was used. Macrophages/TAM were isolated using the anti-F4/80 MicroBeads UltraPure (all Miltenyi Biotec). Cell purity yielded > 95% for T cells, 60 - 95% for macrophages and 75 - 90% for MDSC. For RNA analyses, single cell suspensions were enriched for myeloid cells using the CD11b^+^ MACS kit (Miltenyi Biotec) and stained with Fixable Viability Dye (eBioscience, Frankfurt, Germany), anti-CD45 (clone: 30-F11), anti-CD11b (clone: M1/70), anti-Ly6G (clone: 1A8) and anti-Ly6-C (clone HK1.4; all BioLegend, London, UK) for 30 min on ice. Cells were washed and sorted for CD11b^+^Ly6C^lo^Ly6G^+^ PMN-MDSC or CD11b^+^Ly6C^hi^Ly6G^−^ M-MDSC on a BD FACSAria III (BD Biosciences), yielding mean purities of > 90% (tumor) and > 95% (spleen) (Additional file 1: Figure S3A).

### FACS analysis

Prior to fluorochrome staining, FcRIII/II blocking was performed using the TrueStain fcX™ antibody (Biolegend, London, UK). Cell surface staining was done with anti-CD3 (clone 145-2C11), anti-CD4 (clone GK1.5), anti-CD8 (clone 53–6.7), anti-CD11b (clone M1/70), anti-CD11c (clone N418), anti-CD19 (clone 6D5), anti-CD26 (clone H194–112), anti-CD45 (clone 30-F11), anti-CD69 (clone H1.2F3), anti-CD172a (clone P84), anti-CD206 (clone C068C2), anti-EpCAM (clone G8.8), anti-F4/80 (clone BM8), anti-Ly6C (clone HK1.4), anti-Ly6G (clone 1A8), anti-MHC-I (clone AF6–88.5), anti-MHC-II (clone AF6–120.01), anti-NK1.1 (clone PK136), anti-PD-1 (clone 29F.1A12), anti-PD-L1 (clone 10F.9G2), anti-CD86 (clone GL-1), anti-CD40 (clone 3/23), anti-XCR1 (clone ZET; all BioLegend, London, UK) and anti-CD204 (clone 2F8, Biorad, Munich, Germany) antibodies, and Fixable Viability Dye (Thermo Fisher Scientific, Karlsruhe, Germany) was used to exclude dead cells. The gating strategy is depicted in Additional file 1: Figure S1. Intracellular staining was done for arginase-1 (Polyclonal Sheep IgG; R&D Systems, Minneapolis, USA) using the eBioscience™ FoxP3/Transcription Factor Staining Buffer Kit (Thermo Fisher Scientific, Karlsruhe, Germany). Data were acquired on a BD LSRFortessa system (BD Bioscience, Heidelberg, Germany) and analyzed with FlowJo X software (FLOWJO LLC, Ashland, OR, USA).

### RNA sequencing

RNA from MDSC and tumor tissue was isolated using the QIAzol Lysis buffer together with the RNeasy Kit (Qiagen, Hilden, Germany), according to the manufacturer’s instructions. RNA concentration and rRNA integrity was measured using a Pico 6000 Assay (Agilent Technologies, Ratingen, Germany). RIN values were reached > 7 (Additional file 1: Figure S3A) and total RNA yield was 6.8–350 ng. RNA sequencing library was prepared using the SMARTer® Stranded Total RNA-Seq Kit v2 - Pico-Input Mammalian (Takara, Saint-Germain-en-Laye, France). Briefly, ~ 10 ng RNA was fragmented for 4 min at 94 °C, followed by first-strand cDNA synthesis following adding of Illumina Adapters and Indexes. RNA sequencing library was isolated using AMPure beads, and ribosomal RNA was depleted using ZapRv2 and R-Probes v2. RNAseq library was amplified in 13 cycles and isolated using AMPure beads. Mean tumor cell contamination, as determined by expression of cytokeratin 8 or 18, was < 1%, for tumor-derived MDSC populations (Additional file 1: Figure S3B).

### Bioinformatic data analysis

Quality of sequencing reads was assessed using fastQC (http://www.bioinformatics. babraham.ac.uk/projects/fastqc). Reads were mapped against the mouse genome (mm10) and mouse rRNA sequences with ContextMap version 2.7.9 [32], using BWA [33] as internal short read aligner and allowing at most 4 mismatches per read. Number of read fragments per gene were determined in a strand-specific manner from mapped RNA-Seq reads, using featureCounts [34] and Gencode (v16) annotations. Gene expression was quantified as numbers of fragments per kilobase of transcript per million mapped reads (FPKM). Principal component analysis (PCA) was performed in R for all genes with a median FPKM ≥1, for conditions compared. Differential gene expression analysis was performed on gene read counts using DEseq2 for all genes with an average of 25 reads per sample [35]. *P*-values were adjusted for multiple testing using the method by Benjamini and Hochberg [36], and genes with an adjusted *p*-value < 0.001 and at least a 2-fold change in expression (fold-change ≥2 or log2 fold-change ≤1/2) were considered significantly differentially expressed. The RNA-Seq analysis workflow was implemented and run using the workflow management system Watchdog [37]. Gene set enrichment analysis for all genes ranked by gene expression log2 fold-change was performed using GSEA [38] for MSigDB gene sets (FDR q-value cutoff 0.05):

GSE24102_GRANULOCYSTIC_MDSC_VS_NEUTROPHIL_DN/UP, GSE5099_CLASSICAL_M1_VS_ALTERNATIVE_M2_MACROPHAGE_DN/UP, GSE5099_MONOCYTE_VS_CLASSICAL_M1_MACROPHAGE_DN/UP.

Functional enrichment analysis for up- and downregulated genes was performed using the DAVID webserver [39], against the background of all genes included in the differential gene expression analysis (adj. p-value < 0.01).

### qRT-PCR

Total RNA was isolated using the peqGold TriFast™ Kit (VWR International GmbH, Darmstadt, Germany) according to the manufacturer’s instructions. cDNA synthesis was done with the RevertAID™ First strand cDNA Synthesis kit (Thermo Fisher Scientific, Karlsruhe, Germany) and qRT-PCR was performed with KAPA PROBES FAST qPCR Maser Mix (2x) Kit (Sigma-Aldrich, Taufkirchen, Germany), on the LightCycler 480 II system (Roche Diagnostics, Penzberg, Germany). Primers were designed with the Universal Probes Library.

### T cell suppression assay

For the assessment of suppressive capacity of MDSC or macrophages, a co-culture with T cells was done. For this, 5 × 10^4^ CFSE-labeled T cells (per well) from tumor-naïve C57BL/6 mice were seeded into 96-well plates and co-cultured with 1.25 × 10^4^ (0.25:1), 2.5 × 10^4^ (0.5:1) or 5 × 10^4^ (1:1) MDSC or macrophages. Each well was supplemented with 1 μl anti-CD3/anti-CD28 mAb-coated beads (gibco®, Thermo Fisher Scientific, Karlsruhe, Germany). After 72 h, CFSE dilution of CD4^+^ and CD8^+^ T cells was analyzed by flow cytometry. IFN-γ secretion following co-culture was measured from supernatants at an E:T ratio of 1:1, with ELISA (BD OptEIA, BD Biosciences, Heidelberg, Germany).

### Antigen presentation assay

To assess antigen presentation of MDSC, 5 × 10^4^ CFSE-labeled OT-I T cells were seeded into 96-well plates and co-cultured with 1.25 × 10^4^ (0.25:1), 2.5 × 10^4^ (0.5:1) or 5 × 10^4^ (1:1) MDSC. Tumor-derived MDSC were co-cultured without further treatment. Splenic MDSC were incubated with 1 μg/ml OVA protein over night at 37 °C or loaded with SIINFEKL (100 μg/ml). Subsequently, MDSC were washed and seeded as described above. After 72 h, CFSE dilution of CD8^+^ T cells was analyzed by flow cytometry.

### Statistical analysis

Data present means +/− standard error of the mean (SEM) of biological replicates. Significant differences between two groups were calculated using the Mann Whitney U test or if indicated using an unpaired two-sided students t test. Multiple comparisons were analyzed using Kruskal Wallis test. In case of significant results, subsequent post hoc test was calculated for selected comparisons as indicated. Spearman’s rank-order correlation was performed to analyze associations. To analyze the influence of genotype and treatment a 2-way ANOVA was performed. In case of a significant result, post hoc test between treatments was performed as indicated. Statistical analysis was performed using GraphPad Prism software (version 7.04); *p*-values < 0.05 were considered significant.

## Results

### KPC-derived PDAC is characterized by infiltration with myeloid cells and a T cell-deprived TME

**Fig. 1:**
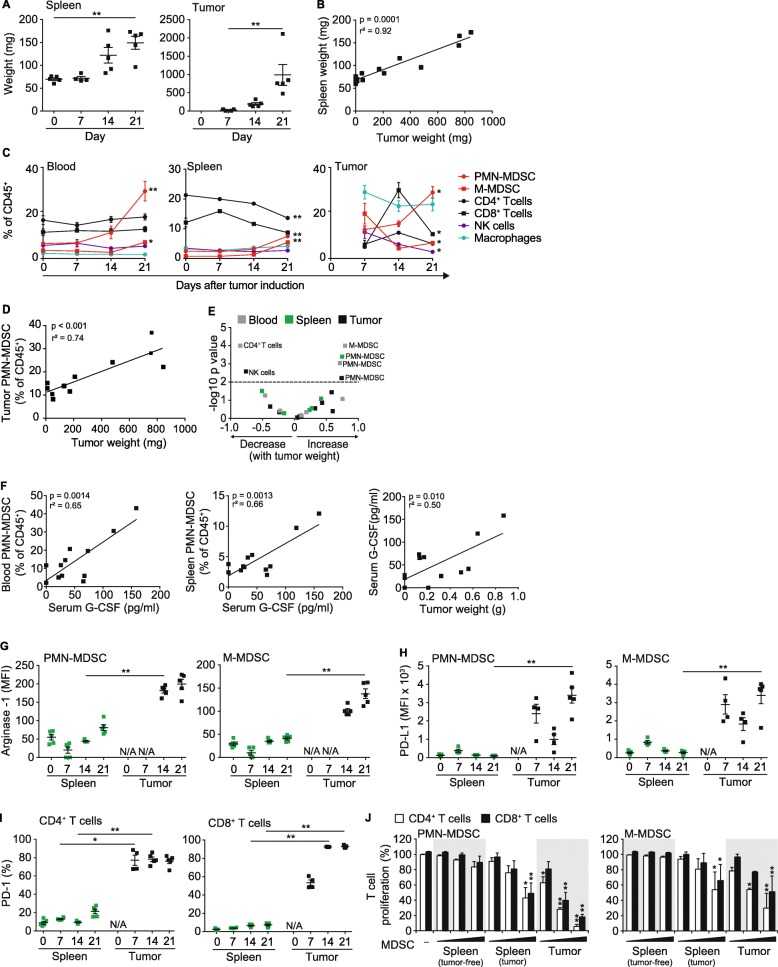
KPC-derived PDAC is characterized by infiltration with myeloid cells and a T cell-deprived tumor microenvironment (TME). T110299 tumors were implanted orthotopically in syngeneic C57BL/6 mice which were sacrificed at days 7, 14 and 21 after tumor induction for analysis of blood, spleen and tumor. **a-b** Spleen and tumor weights and respective correlation analysis. **c** Relative frequency of leukocytes in blood, spleen and tumor. **d-e** Correlation of relative immune cell frequency with tumor weight. **f** Correlation of serum G-CSF level with PMN-MDSC frequency in blood and spleen as well as correlation of tumor weight with G-CSF level in serum. **g-h** Surface expression of arginase-1 and PD-L1 on MDSC. **i** PD-1 expression on T cells in spleens and tumors. **j** MDSC-like cells from naïve mice as well as MDSC from spleens and tumors of tumor-bearing mice were isolated and co-cultured with CFSE-labeled T cells in increasing effector (E; MDSC) to target (T; T cell) ratios (E:T) of 0.25:1, 0.5:1 and 1:1, in the presence of anti-CD3/anti-CD28 mAb-coated beads. After 72 h CFSE dilution of T cell populations was assessed. **a**,**c**,**g**,**h**,**i** Data ± SEM is shown for *n* = 4–5 mice per group. **b**,**d**,**f** *n* = 12 mice (**e**) n = 12 mice / group (**c**) Statistics for the comparison of day 0 and day 21 (blood and spleen), and day 7 and day 21 (tumor) are shown. (**j**) Representative graph of three independent experiments, Data± SEM for *n* = 2 mice per group, unpaired two-sided students t test (*p < 0.05, **p < 0.01, in J compared to tumor-free control)

The KPC-derived T110299 PDAC mouse model shares many pathological features observed in human disease. As such, we investigated the impact of T110299 tumors on myelopoiesis, the TME and its immune cell composition. Tumor cells were implanted into the pancreas of syngeneic C57BL/6 mice and subsequently, immune cell composition in blood, spleen and tumors was monitored within 21 days of tumor development. Tumor engraftment was evident within the first week and progressed rapidly during the following 2 weeks. Tumor growth was paralleled by splenomegaly without any signs of metastasis, indicating influx or proliferation of hematopoietic cells (Fig. 1a-b). Analysis of immune cell composition (Additional file 1: Figure S1) during tumor progression revealed an expansion of myeloid cells in blood, spleen and tumor. The expansion of the myeloid compartment was most pronounced in tumor tissue and identified as CD11b^+^Ly6C^int^Ly6G^+^ PMN-MDSC and CD11b^+^Ly6C^hi^Ly6G^−^ M-MDSC, as well as CD11b^+^Ly6C^low/int^ F4/80^+^ macrophages, as major cell populations (Fig. 1c). In tumors, myeloid cell recruitment (macrophages, PMN-MDSC and M-MDSC) preceded T cell infiltration, with T cells transiently peaking on day 14. On day 21, the immune cell infiltrate was dominated by macrophages and PMN-MDSC. Correlation analysis further revealed a strong correlation between tumor size and PMN-MDSC expansion, both systemically and in tumor tissue (Fig. 1d). Overall, we predominantly observed increased PMN-MDSC populations with increased tumor weight in blood, spleen and tumor, whereas blood CD4^+^ T cells as well as tumor-resident NK cells decreased (Fig. 1e). We also investigated in more detail the role of the growth factor G-CSF, which is produced by KPC-derived PDAC and known to induce proliferation of granulocytic precursor cells in tumor bearing hosts [13]. In our PDAC model, serum levels of G-CSF were increased in tumor-bearing mice, and highly correlated with PMN-MDSC populations in blood and spleen, as well as with tumor weight, suggesting G-CSF to be a major driver for the expansion of PMN-MSC with a strong immunosuppressive phenotype (Fig. 1f).

The inverse correlation of MDSC and T cell infiltration during PDAC progression prompted us to characterize immune suppressive mechanisms of the TME. Within the MDSC compartment, we investigated the expression of known immune suppressive mediators, such as arginase-1 and the checkpoint molecule PD-L1. Arginase-1 levels were comparably low in splenic PMN- and M-MDSC during tumor development, but highly upregulated in tumor-resident MDSC (Fig. 1g). Similar characteristics were also found for PD-L1 expression (Fig. 1h). Moreover, the PD-L1 counterpart, PD-1, was expressed on the vast majority of tumor-resident CD8^+^ and CD4^+^ T cells (Fig. 1i). Subsequently, we assessed the ability of MDSC to inhibit T cell activation, which is the population-defining hallmark of MDSC. We isolated MDSC populations from spleen and tumors to set up a co-culture with anti-CD3/CD28 mAb-activated T cells from tumor-free mice, and used monocytes and granulocytes isolated from spleens of tumor-free mice as controls. Only MDSC from PDAC-bearing mice displayed pronounced suppressive effects on CD8^+^ as well as CD4^+^ T cell proliferation. Whilst PMN-MDSC turned out to be more suppressive than M-MDSC, overall MDSC populations isolated from tumors exhibited the most pronounced suppressive capacity (Fig. 1j). Together, the data show that KPC-derived PDAC develop typical features of a suppressive TME characterized by pathologically activated myeloid cells with high suppressive capacity.

### Poly(I:C)_c_ reduces tumor mass in PDAC concomitant with enhanced T cell activation and reduced suppressive capacity of MDSC

**Fig. 2:**
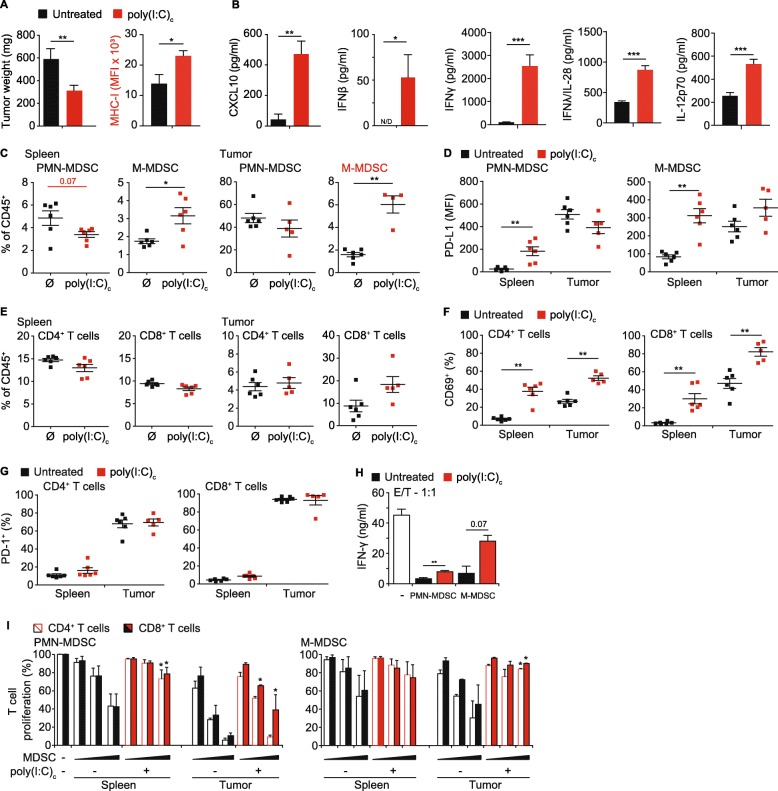
Poly(I:C)_c_ reduces tumor mass in PDAC concomitant with enhanced T cell activation and reduced suppressive capacity of MDSC. Mice with orthotopic T110299 tumors were treated with poly(I:C)_c_ twice prior to sacrifice at day 21 after tumor induction. **a** Tumor weights, tumor cell MHC-I expression and (**b**) serum cytokine levels. **c** Frequencies of MDSC populations in spleen and tumor of untreated and poly(I:C)_c_-treated mice. **d** Surface expression profiles of PD-L1 on MDSC subsets. **e** Frequencies of T cell populations in spleen and tumor of untreated and poly(I:C)_c_-treated mice. **f-g** CD69 and PD-1 surface expression of splenic and tumor-resident T cells. **h** Representative data of IFN-γ secretion in MDSC / T cell co-cultures, at a ratio of 1:1, following anti-CD3/anti-CD28 mAb-coated beads stimulation for 72 h. **i** Splenic T cells and MDSC from spleens and tumors of untreated or poly(I:C)_c_-treated tumor-bearing mice were isolated and co-cultured with CFSE-labeled T cells in increasing effector (E; MDSC) to target (T; T cell) ratios (E:T) of 0.25:1, 0.5:1 and 1:1 in the presence of anti-CD3/anti-CD28 mAb-coated beads. After 72 h CFSE dilution of CD4^+^ and CD8^+^ T cells was assessed. **a-f** Data ± SEM is shown for *n* = 5 to 8 mice per group. **g**-**h** Representative graph of three independent experiments. Data± SEM for n = 2 mice per group,unpaired two-sided students t test (*p < 0.05; **p < 0.01)

We showed earlier that a systemic therapy with the MDA5 ligand poly(I:C)_c_ had a positive effect on survival of PDAC-bearing mice, which was dependent on the presence of cytotoxic T cells [27]. Other studies with RLH ligands pointed towards a reduced number or altered function of MDSC in treated animals [24, 28, 40]. To study the effect of RLH activation on MDSC in fully established tumors in more detail, we treated mice with poly(I:C)_c_ i.v. and analyzed the tumors 21 days after tumor induction. Treatment resulted in a 50% reduction in tumor mass (Fig. 2a). Downregulation of MHC class I is a common mechanism of tumors to evade the immune system. We could previously show that stimulation of pancreatic cancer cells in vitro with RLH ligands induces the up-regulation of MHC-I as well as CD95 (Fas), resulting in more effective tumor cell killing by cytotoxic T cells [26]. In line with these in vitro findings, poly(I:C)_c_ led to a profound upregulation of MHC-I molecules on tumor cells in vivo (Fig. 2a). RLH ligands are strong inducers of type I IFN, which in turn mounts a robust immune response connecting innate with adaptive immunity. As such, RLH treatment resulted in high levels of CXCL10 and IFN-β, which were accompanied by T_h_1-supporting IL-12p70 and IFN-y as well as IL-28, an important type III IFN further supporting CTL-mediated cytotoxicity (Fig. 2b).

We observed a relative reduction of PMN-MDSC, whereas M-MDSC frequency increased in both spleen and tumor (Fig. 2c). Analysis of MDSC surface marker expression revealed a strong therapy-mediated PD-L1 induction in spleen. Similar to our earlier observations in treatment-naïve mice, we found high basal PD-L1 expression by MDSC within the tumor tissue, which was not further increased by immunotherapy (Fig. 2d). T cell frequencies in spleens were unaltered; however, increased infiltration of CD8^+^ T cells was detected within the tumor tissue, which is in line with our previous observations (Fig. 2e). Both splenic and tumor-infiltrating T cells upregulated expression of the early activation marker CD69 in response to poly(I:C)_c_, whereas PD-1 expression was unaffected (Fig. 2f-g). We then assessed whether poly(I:C)_c_ treatment affects the suppressive capacity of MDSC. We isolated MDSC populations from spleen and tumor of untreated and treated PDAC-bearing mice and studied their influence on T cell proliferation. In order to assess overall immunosuppressive effects of MDSC T cell response, we measured IFN-γ secretion as key cytokine of T cell activation in MDSC co-cultures. As expected, at an effector (MDSC) to target (T cell) ratio of 1:1, IFN-γ secretion was strongly suppressed by MDSC (Fig. 2h); however, this was - at least in parts - rescued in co-cultures with MDSC from mice previously treated with poly(I:C)_c_. As observed before, tumor-derived MDSC were more suppressive compared to their splenic counterparts, with the highest suppression seen in PMN-MDSC co-cultures, for both CD8^+^ and CD4^+^ T cells. Suppressive function of MDSC populations from poly(I:C)_c_-treated animals was reduced for both, PMN-MDSC and M-MDSC (Fig. 2i). These findings are indicative of a functional in vivo reprogramming of MDSC in poly(I:C)_c_-treated mice.

Analysis of splenic and tumor-resident B and NK cells showed a slight increase in splenic B cell numbers; both cell populations upregulated CD69 expression upon therapy (Additional file 1: Figure S2A-B). Poly(I:C)_c_ treatment increased both the intratumoral frequency of migratory cross-presenting conventional DC 1 (cDC1) as well as their activation measured by CD40 expression. In addition, the co-stimulatory molecule CD86 was upregulated in both CD11c^+^MHC-II^hi^CD26^+^XCR1^+^CD172a^−^ cDC1 and CD11c^+^MHC-II^hi^CD26^+^CD172a^+^XCR1^−^ cDC2 in the tumor-draining lymph node (Additional file 1: Figure S2C-D). Interestingly, the relative frequency of macrophages/TAM was significantly reduced in treated animals, in both spleen and tumor. Moreover, macrophages/TAM showed an activated phenotype with enhanced MHC-I expression (Additional file 1: Figure S2E-G). Further analyses revealed that the frequency of M2-like CD204^+^CD206^+^ macrophages, known to highly correlate with poor disease outcome in patients with various cancer types [41–43], was decreased in tumors (Additional file 1: Figure S2E-F). TAM showed a strong T cell suppressive phenotype, which was – in contrast to MDSC – not reversed upon poly(I:C)_c_ treatment (Additional file 1: Figure S2H).

### Transcriptomic profiling reveals a therapy-induced reprogramming of MDSC

**Fig. 3:**
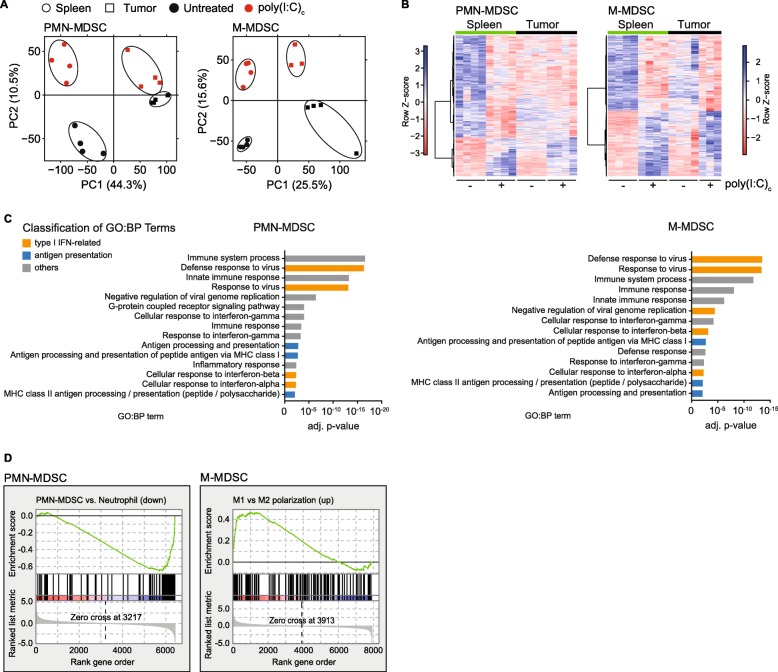
Poly(I:C)_c_ triggers transcriptional reprogramming of MDSC. Mice with orthotopic T110299 tumors were treated twice with poly(I:C)_c_ prior to sacrifice as described before. RNA of MDSC populations was isolated for whole transcriptome analysis. **a** Principal component analysis (PCA) of transcriptome of splenic or tumor-derived MDSC with and without poly(I:C)_c_ treatment. **b** Heatmap of gene expression values (colors indicate row z-scores) for the 1.000 genes contributing most to principle component 2 (PC2). **c** DAVID analysis for enriched gene ontology biological processes (GO:BP) terms from differentially expressed genes (adjusted *p* < 0.001, ≥ 2-fold change) upon poly(I:C)_c_ treatment from splenic MDSC. **d** Gene set enrichment analysis (GSEA) of differentially expressed genes upon poly(I:C)_c_ treatment compared to published gene sets describing PMN-MDSC vs. neutrophils (GSE24102) and macrophage polarization (GSE5099). Data shown for *n* = 3 to 4 mice per group

To better understand the mechanisms by which MDSC undergo phenotypical changes upon systemic immunotherapy, we performed a whole transcriptome analysis of PMN- and M-MDSC populations from spleens and tumors. Mice with orthotopic PDAC were treated on day 18 and 20 after tumor implantation with poly(I:C)_c_ or were left untreated. On day 21, MDSC were sorted for high purity **(**Additional file 1: Figure S3A-B), followed by RNA extraction and next-generation sequencing. An unbiased principle component analysis (PCA), using the ~ 14.000 most expressed genes, was performed. For both PMN- and M-MDSC, the replicates of each condition clustered closely, confirming the high quality of the data. PCA revealed that PC1 distinguishes samples based on the compartment they were isolated from (PMN-MDSC: 44.3%; M-MDSC: 25.5%), and PC2 described the changes that were induced by poly(I:C)_c_ treatment (PMN-MDSC: 10.5%; M-MDSC: 15.6%) (Fig. 3a). The 1000 genes contributing most to PC2 in PMN- and M-MDSC show a similar regulation in both spleen and tumor (Fig. 3b).

The treatment-induced transcriptomic changes were analyzed by a differential gene expression analysis (adjusted *p* < 0.001, ≥ 2-fold change) in PMN-MDSC (spleen: 420; tumor: 180; shared: 100) and M-MDSC (spleen: 584; tumor: 210; shared: 113) **(**Additional file 1: Figure S3C). Functional annotation analysis using the Database for Annotation, Visualization and Integrated Discovery (DAVID) was done with differentially expressed genes from spleen. Genes were found to be significantly enriched in gene ontology biological process (GO:BP) clusters related to immune system processes, virus and IFN response-related pathways, and antigen presentation-related genes (Fig. 3c and Additional file 1: Figure S4). Most importantly, gene set enrichment analysis of splenic differentially expressed genes revealed an enrichment of neutrophil-associated gene signature for PMN-MDSC and enrichment of M1-associated genes for M-MDSC after poly(I:C)_c_ therapy, suggesting the phenotypic reprogramming of MDSC **(**Fig. 3d**)**.

### MDSC of treated mice do not acquire professional antigen presenting cell function

**Fig. 4:**
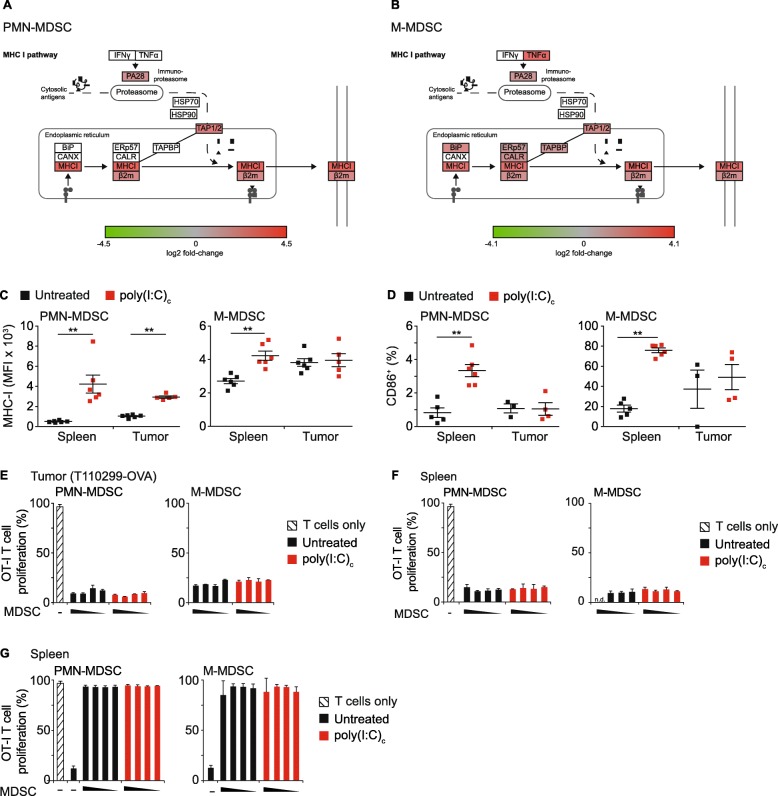
MDSC of treated mice do not acquire professional antigen presenting cell function. **a-b** Schematic representation of differential gene expression upon poly(I:C)_c_ treatment annotated in the KEGG pathway antigen processing and presentation of PMN- and M-MDSC. **c-g** Mice with orthotopic ovalbumin expressing PDAC (T112099-OVA) were treated with poly(I:C)_c_ twice prior to sacrifice at day 21 after tumor implantation. **c-d** Surface expression of MHC-I and CD86^+^ of MDSC populations at baseline and upon poly(I:C)_c_ treatment. **e-g** MDSC from (**e**) tumor and (**f-g**) spleen of untreated or poly(I:C)_c_-treated tumor-bearing mice were isolated. Splenic MDSC were either treated with OVA protein (**f**) or SIINFEKL peptide (**g**). Subsequently MDSC were co-cultured with CFSE-labelled OT-I T cells with an increasing effector (E; MDSC) to target (T; T cell) ratio (E:T) of 0.25:1, 0.5:1 and 1:1 and CFSE dilution of CD8^+^ T cells was assessed following 72 h of co-culture. **c-d** Data are shown for n = 5 to 6 mice per group. **e-g** Representative graph of two independent experiments, Data± SEM for n = 2 mice per group (n.d. = not determined; *p < 0.05; **p < 0.01)

One of the significantly enriched gene clusters was associated with MHC class-I antigen presentation. In both PMN- and M-MDSC, essential components of the MHC-I-dependent antigen processing and presentation machinery, including the immunoproteasome, the peptide transporter TAP and the MHC-I complex, were up-regulated following poly(I:C)_c_ therapy (Fig. 4a-b). Flow cytometric analysis revealed a therapy-induced upregulation of MHC-I expression for PMN-MDSC in spleen and tumor, and for M-MDSC in spleen only (Fig. 4c). Moreover, upregulation of the costimulatory molecule CD86 was observed in a subset of splenic PMN-MDSC and the majority of M-MDSC. Tumor-resident M-MDSC already expressed high levels of CD86 and remained unaltered upon therapy (Fig. 4d).

To investigate the ability of MDSC to present tumor-associated antigen on MHC-I, ovalbumin (OVA)-expressing T110299 tumors (T110299-OVA) were used as model. 18 and 20 days after tumor induction, mice were treated with poly(I:C)_c_ or left untreated and MDSC from both tumor and spleen were isolated. Tumor-derived PMN- and M-MDSC were unable to induce antigen-dependent CD8^+^ T cell proliferation, irrespective of treatment (Fig. 4e). In Addition, we evaluated the capability of MDSC to process and cross-present OVA protein ex vivo. Splenic MDSC from T110299-OVA tumor-bearing hosts were incubated overnight with OVA protein and subsequently co-cultured with OT-I T cells for 3 days. Again, no T cell proliferation was detectable (Fig. 4f). To rule out that the lack of functional cross-presentation is due to T cell inhibition by MDSC, the presentation of exogenously added SIINFEKL peptide was assessed. For this, MDSC of T110299-OVA tumor bearing hosts were isolated, pulsed with SIINFEKL peptide and subsequently co-cultured with OT-I T cells. Peptide-loaded MDSC were able to induce a strong OT-I T cell proliferation, with no detectable differences between MDSC of untreated or treated mice (Fig. 4g). Together, these data rule out a function of MDSC as professional antigen presenting cells, which was irrespective of their polarization status.

### Therapeutic efficacy and immune activation of MDA5-targeted immunotherapy is mediated by type I IFN signaling

MDA5 activation is known to induce type I IFN and the transcriptomic profile of MDSC in poly(I:C)_c_ treated mice confirmed a predominant type I IFN response. To further evaluate the role of IFN signaling on MDSC function and tumor control, therapeutic efficacy of poly(I:C)_c_ treatment was assessed in PDAC-bearing wild-type and IFNAR1-deficient mice.

**Fig. 5:**
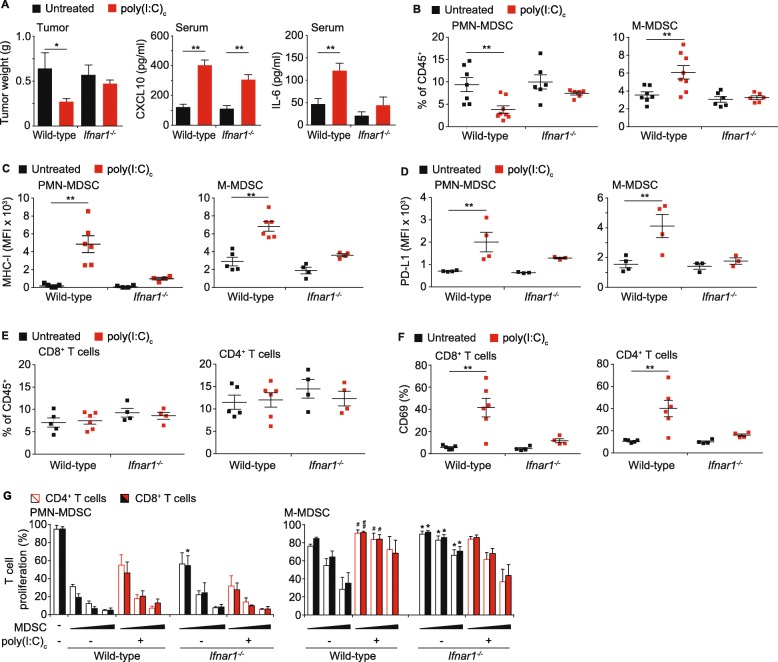
Bidirectional control of MDSC suppressive function by type I interferon signaling in PDAC. Wild type and IFNAR1-deficient mice were transplanted with T110299 orthotopic tumors and treated with poly(I:C)_c_ twice prior to sacrifice at day 21 after tumor induction. **a** Tumor weights, CXCL10 and IL-6 serum levels in untreated and treated mice. **b** Splenic MDSC frequency. **c-d** MHC-I and PD-L1 surface expression on splenic MDSC. **e-f** T cell frequency and CD69 expression on splenic T cells. **g** Splenic T cells from untreated C57BL/6 mice and MDSC from spleens of T110299 tumor-bearing wild type or IFNAR1-deficient mice were isolated and T cell suppression was analyzed ex vivo. T cells were co-cultured with an increasing effector (E; MDSC) to target (T; T cell) ratio (E:T) of 0.25:1, 0.5:1 and 1:1 for 72 h in the presence of anti-CD3/anti-CD28 mAb-coated beads. CFSE dilution of CD4^+^ and CD8^+^ T cell populations was assessed. **a-f** Data ± SEM is shown for n = 4 to 7 mice per group. **g** Data± SEM for n = 3–5 mice per group, unpaired two-sided students t test(*p < 0.05; **p < 0.01, (**g**) comparison of untreated wild type and untreated IFNAR1-deficient are depicted with *; #*p* < 0.05, ##*p* < 0.01, comparison of untreated wild type and treated wild type are depicted with #)

Tumor weight was significantly decreased in wild-type mice after poly(I:C)_c_ treatment, whereas no difference was observed in *Ifnar1*^*−/−*^ mice, supporting a role of IFN signaling as a prerequisite for anti-tumor efficacy **(**Fig. 5a**)**. As expected, CXCL10 serum levels of both wild-type and *Ifnar1*^*−/−*^ mice were comparable after treatment; however, IL-6 serum levels were significantly decreased in *Ifnar1*^*−/−*^ mice **(**Fig. 5a**)**. Untreated mice had comparable frequencies of MDSC and poly(I:C)_c_ treatment led to a decrease of PMN-MDSC and an increase of M-MDSC numbers in wild-type mice, but not *Ifnar1*^−/−^ mice **(**Fig. 5b**).** Furthermore, MDSC from IFNAR1-deficient mice failed to upregulate MHC-I and PD-L1 expression upon therapy, indicating a critical role for IFN signaling on MDSC numbers and phenotype upon MDA5-based immunotherapy **(**Fig. 5c-d**)**. Neither the genotype nor the treatment had an influence on CD4^+^ and CD8^+^ T cell frequencies in spleen and tumor; however, poly(I:C)_c_ failed to induce CD69 expression in T cells of *Ifnar1*^−/−^ mice (Fig. 5e-f).

Our data show that MDA5-based immunotherapy in PDAC-bearing mice led to a reduction of the suppressive function of MDSC populations, concomitant with a dominant IFN signature in their transcriptomic profile. We therefore investigated the role of type I IFN signaling on the suppressive capacity of MDSC in *Ifnar1*^*−/−*^ mice. Interestingly, in untreated tumor-bearing hosts the suppressive capacity of MDSC was reduced in *Ifnar1*^*−/−*^ mice, as compared to their wild-type controls, pointing towards a role for IFN signaling in early MDSC differentiation into a suppressive phenotype **(**Fig. 5g**)**. Of note, while poly(I:C)_c_ treatment reversed the suppressive capacity of MDSC in wild-type mice, the T cell suppressive function of both PMN- and M-MDSC from *Ifnar1*^*−/−*^ mice was not significantly changed, arguing for a role of IFN signaling in regulating the suppressive function upon MDA5-based therapy.

## Discussion

PDAC remains poorly responsive to many therapies and one major hurdle is the immunosuppressive TME that is created during PDAC progression [2]. MDSC have attracted the field of tumor immunotherapy and are accepted as important factors in shaping the TME. MDSC actively contribute to the TME to preserve an immunologically compromised state. Due to their plasticity, targeting MDSC is difficult and strategies mainly focused on altering recruitment and function [21]. Human and mouse MDSC share similar features, which underlines the importance of translational mouse models as important source to develop novel targeting approaches.

We made use of an KPC-derived orthotopic pancreatic cancer model [44] and show that PDAC develop an immunosuppressive TME characterized by dense infiltration with MDSC and sparse T cell recruitment. During tumor growth, the frequency of PMN-MDSC increased systemically. Similar as observed for human disease, MDSC showed a pathologic activation with enhanced levels of arginase-1 and PD-L1, high T cell suppressive capacity, and compartmentalized differences accentuating tumor-resident MDSC with increased suppressive activity [17, 45]. Using the KPC-derived pancreatic cancer model, we observed a more potent suppressive capacity of PMN-MDSC as compared to M-MDSC. The pathological activation of PMN-MDSC is a hallmark of KPC-derived pancreatic cancer, a finding that is covered by a recent report of Li et al. in a similar tumor model, demonstrating PMN-MDSC as key determinants of the immunosuppressive TME [46]. The literature provides evidence for both MDSC populations bearing strong suppressive capacity [47–49].

During tumor progression, the tumor induces a highly complex secretome, which is characteristic for the TME and for entertaining the suppressive phenotype by accelerated myelopoiesis, impaired differentiation, and enhanced pathological activation of MDSC. In PDAC the TME is characterized by high levels of growth factors (e.g. G-CSF, GM-CSF) and cytokines (e.g. IL-6, TGF-β), accounting for the chronic inflammatory and suppressive phenotype [50]. It is conceivable that such tumor-derived signals are able to program myeloid cells towards an tumor-promoting phenotype, with systemic effects targeting spleen and bone marrow. A crucial factor involved in the generation and pathological activation of PMN-MDSC is the growth factor G-CSF, and systemic levels of G-CSF have been correlated with MDSC accumulation in several tumor models [46, 51, 52]. We speculate that G-CSF also accounts for the pathological activation of PMN-MDSC in KPC-derived pancreatic cancer, as elevated G-CSF serum levels during tumor progression correlated with the frequency of PMN-MDSC in spleen and blood. Thus, blocking G-CSF signaling could provide a means to alleviate MDSC-mediated immune suppression.

Preclinical data and early clinical trials showed that preventing the accumulation of PMN- and M-MDSC by blocking the CXCR2- and CCR2-dependent migration, respectively, might serve as strategy to change the immunosuppressive TME [53]. We sought to investigate the effects of an RLH-based immunotherapy as approach for reprogramming an immunosuppressive into a therapy-vulnerable “hot” TME [11]. We could earlier show that mimicking a viral infection by the injection of synthetic RLH ligands augmented anti-tumor immunity and greatly induced an immunogenic form of tumor cell death [26]. The RLH-targeting therapy broadly altered the cellular immune landscape in spleens and tumors, which also included alterations within MDSC populations [28]. We previously reported that the MDA5-based therapeutic efficacy in PDAC is mediated by CD8^+^ T cells [27]. Here, we show that systemic T cell activation and concomitant tumor reduction are dependent on intact IFNAR signaling. This has also been confirmed in melanoma studies showing that both lymphoid and myeloid IFNAR signaling is critical for the therapy response, underlining the central role for IFN in anti-tumor immunity [54, 55].

We found that the change in peripheral MDSC frequency upon therapy was dependent on IFNAR1 signaling. An IFN-mediated increase of M-MDSC and decrease of PMN-MDSC frequency has recently been described in the context of chronic CMV infection, which was linked to an IFN-mediated induction of IRF8 expression in myeloid precursor cells [23]. The expression of MHC-I and PD-L1 were markedly elevated upon poly(I:C)_c_ treatment, but greatly reduced in IFNAR-deficient mice. This is not surprising, as MHC-I and PD-L1 belong to the group of IFN-stimulated genes, which is in line with a recent report showing IFN as inducer of PD-L1 on MDSC [56]. However, the observed IFNAR1-dependent alterations in immune cell activation, which also included a profound T cell activation, are characteristic for the transition of a “cold” towards a “hot” tumor. Antigen presentation and T cell priming play a central role in anti-tumor immunity and IFN is required for efficient cross-presentation by DC. Immune escape mechanisms, such as downregulated MHC-I expression, are frequently used by tumors to evade immune responses. The expression of MHC-I molecules is a crucial event in engaging tumor-reactive T cells, and we confirmed upregulated MHC-I expression on tumors following poly(I:C)_c_ treatment. We saw a strong therapy-induced decrease of TAM, which - similar to MDSC - mediated profound T cell inhibition in vitro. Furthermore, therapy decreased M2 polarization of TAM. However, in contrast to MDSC, the suppressive phenotype of TAM was not altered by poly(I:C)_c_ therapy. Genes associated with antigen presentation were upregulated upon poly(I:C)_c_ treatment, and there are several reports of immunotherapeutic strategies that induce antigen presentation by MDSC, including TLR agonists [40]. In our model, poly(I:C)_c_ was used as MDA5-specific agonist and we cannot rule out potential TLR3 engagement; nevertheless, we did not observe cross-presentation of tumor-associated antigen by MDSC.

Low and chronic IFN signaling is observed in tumor-bearing hosts and has been linked to support the immunosuppressive network [54]. Similar observations have been made for other chronic disease models, such as Western diet-induced atherosclerosis or viral infections, in which chronic inflammation is accompanied by a type I IFN signature [57, 58]. In line with this, we compared the transcriptomic profiling of PDAC bulk tumors with normal pancreas tissue and confirmed an upregulated cellular response to IFN-β (adj. *p* < 0.01) (Additional file 1: Figure S3D).

There is evidence that IFN can also repolarize neutrophils and macrophages to an anti-tumor phenotype [20, 59], which is in agreement with observations made for MDSC in terms of TLR7/8- and TLR9-targeted therapies [24, 40]. Both, the TLR and MDA5 signaling cascades, lead to the activation of a shared IRF3/7 signaling pathway, with the induction of proinflammatory cytokines and type I IFN. RLH-signaling also induces proinflammatory cytokines via the NF-κB pathway consequentially upregulating CXCL10 and IL-6 levels. Interestingly, we observed reduced IL-6 levels in IFNAR1-deficient mice pointing towards a role for IFN signaling in regulating inflammation. IFNAR signaling has been shown to amplify early proinflammatory cytokine production during virus infection [60]; therefore, it is conceivable that IFN and cytokine signaling mutually act on anti-tumor immunity. We found that poly(I:C)_c_ treatment reduced the suppressive capacity of MDSC populations in wild-type mice, but not in IFNAR1-deficient mice. Of note, steady-state suppressive capacity of MDSC was significantly less-pronounces in IFNAR1-deficient hosts; thus, it is tempting to speculate that early- and late-stage IFN signaling share a causal relationship in MDSC development and tumor control.

## Conclusions

This study provides an in-depth analysis of MDSC in RLH-based immunotherapy using a state-of-the-art genetic model of pancreatic cancer. Our systematic approach and comprehensive analysis of MDSC provide an interface of cell-specific transcriptomic data analysis and cancer immunology. Our work supports a rationale for RLH-ligands as promising combination partners for other immune-based strategies, including chemo- or radiotherapy, checkpoint inhibition or CAR-T cells. Thus, combination therapies might benefit from the remodeling capacities of the RLH-based immunotherapy, to achieve greater clinical improvements.

## Supplementary information

**Additional file 1: Figure S1.** Gating strategy for the identification of MDSC populations. **Figure S2.** Poly(I:C)_c_ reduces macrophage frequency and activates macrophages, cDC, B and NK cells. **Figure S3.** Poly(I:C)_c_ triggers transcriptional reprogramming of MDSC. **Figure S4.** Significantly regulated genes in PMN- and M-MDSC upon poly(I:C)_c_ therapy.

## Data Availability

The datasets generated and/or analyzed during the current study are available in the Gene Expression Omninus (GEO) repository with the accession number GSE126879, https://www.ncbi.nlm.nih.gov/geo/query/acc.cgi?acc=GSE126879; Until publication, enter token “mrcrekiwpnippsp” into the box to access data.

## Abbreviations

ATRA: all-trans retinoic acid
CAR: Chimeric antigen receptor
CCR2: Chemokine (C-C motif) receptor 2
cDC: conventional dendritic cell
CXCR2: Chemokine (C-X-C motif) receptor 2
FDA: Food and Drug Administration
G-CSF: Granulocyte colony-stimulating factor
GM-CSF: Granulocyte-macrophage colony-stimulating factor
IFN: Interferon
IFNAR1: IFN alpha and beta receptor 1
IRF: Interferon regulatory factor
MDA5: Melanoma differentiation-associated protein 5
MDSC: Myeloid-derived suppressor cells
MHC: Major histocompatibility complex
M-MDSC: Monocytic MDSC
OVA: Ovalbumin
PCA: Principle component analysis
PDAC: Pancreatic ductal adenocarcinoma
PD-L1: Programmed cell death ligand 1
PMN-MDSC: Polymorphonuclear MDSC
RIG-I: Retinoic acid inducible gene I
RLH: RIG-I-like helicases
TAM: Tumor associated macrophages
TAN: Tumor-associated neutrophils
TAP: Transporter associated with antigen processing
TGF: Transforming growth factor
TLR: Toll-like receptor
TME: Tumor microenvironment
