# Correction to: Immunostimulatory RNA leads to functional reprogramming of myeloid-derived suppressor cells in pancreatic cancer

**DOI:** 10.1186/s40425-019-0830-7

**Published:** 2019-12-16

**Authors:** Philipp Metzger, Sabrina V. Kirchleitner, Michael Kluge, Lars M. Koenig, Christine Hörth, Carlotta A. Rambuscheck, Daniel Böhmer, Julia Ahlfeld, Sebastian Kobold, Caroline C. Friedel, Stefan Endres, Max Schnurr, Peter Duewell

**Affiliations:** 1 Center of Integrated Protein Science Munich (CIPSM) and Division of Clinical Pharmacology, Klinikum der Universität München, Lindwurmstrasse 2a, 80337 Munich, Germany; 2 Department of Neurosurgery, University Hospital, LMU Munich, 81377 Munich, Germany; 3 Institute for Informatics, Ludwig-Maximilians-Universität München, 80333 Munich, Germany; 4 Institute of Innate Immunity, University of Bonn, Venusberg-Campus 1, 53127 Bonn, Germany

**Correction to: J ImmunoTher Cancer (2019) 7:288**

**https://doi.org/10.1186/s40425-019-0778-7**

Following publication of the original article [1], the authors have reported that Fig. 2 and Additional file 1: Figure S1, S2 partially show red scripts.

**Fig. 2:**
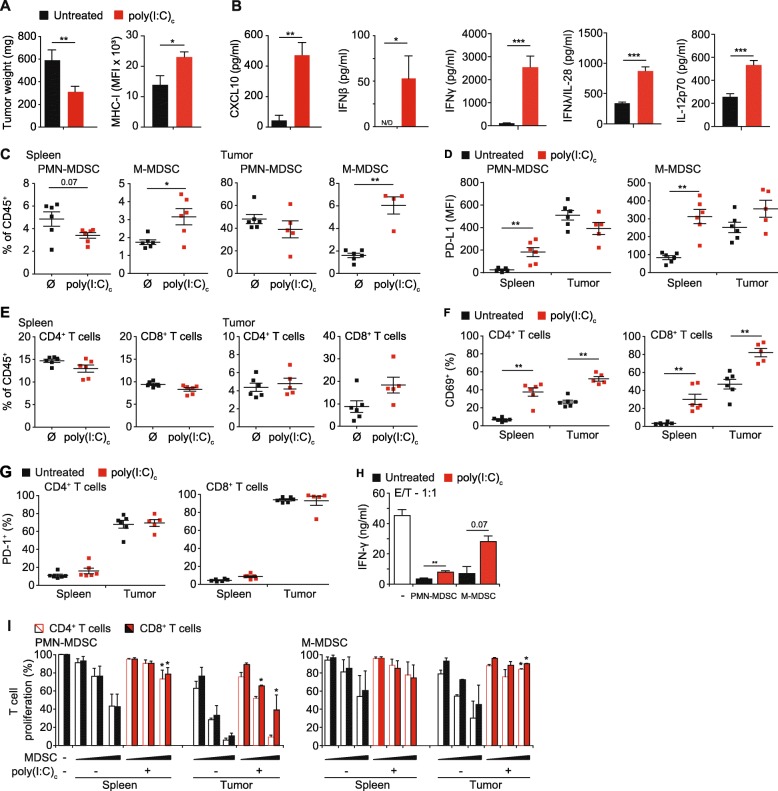
Poly(I:C)_c_ reduces tumor mass in PDAC concomitant with enhanced T cell activation and reduced suppressive capacity of MDSC. Mice with orthotopic T110299 tumors were treated with poly(I:C)_c_ twice prior to sacrifice at day 21 after tumor induction. **a** Tumor weights, tumor cell MHC-I expression and (**b**) serum cytokine levels. **c** Frequencies of MDSC populations in spleen and tumor of untreated and poly(I:C)_c_-treated mice. **d** Surface expression profiles of PD-L1 on MDSC subsets. **e** Frequencies of T cell populations in spleen and tumor of untreated and poly(I:C)_c_-treated mice. **f-g** CD69 and PD-1 surface expression of splenic and tumor-resident T cells. **h** Representative data of IFN-γ secretion in MDSC / T cell co-cultures, at a ratio of 1:1, following anti-CD3/anti-CD28 mAb-coated beads stimulation for 72 h. **i** Splenic T cells and MDSC from spleens and tumors of untreated or poly(I:C)_c_-treated tumor-bearing mice were isolated and co-cultured with CFSE-labeled T cells in increasing effector (E; MDSC) to target (T; T cell) ratios (E:T) of 0.25:1, 0.5:1 and 1:1 in the presence of anti-CD3/anti-CD28 mAb-coated beads. After 72 h CFSE dilution of CD4^+^ and CD8^+^ T cells was assessed. **a-f** Data ± SEM is shown for *n* = 5 to 8 mice per group. **g**-**h** Representative graph of three independent experiments. Data± SEM for *n* = 2 mice per group,unpaired two-sided students t test (*p < 0.05; **p < 0.01)

In Fig. 2:

- A: Red font “MHC-I (MFI x 10^3^)” should change to black font;
- C: Red font “0.07” and “M-MDSC” should change to black font.

In Additional file 1: Figure S1, S2:

- S1: Red font “Spleen” and “Tumor” should change to black font;
- S2: Red font “C”, “D”, “E”, “F”, “G”, “H” should change to black font.

The correct version of the figures can be found below.

The corrections have been implemented in the original article as well.

We apologize for the inconvenience.

## Supplementary information

**Additional file 1: Figure S1.**. Gating strategy for the identification of MDSC populations. **Figure S2.** Poly(I:C)c reduces macrophage frequency and activates macrophages, cDC, B and NK cells. **Figure S3.** Poly(I:C)c triggers transcriptional reprogramming of MDSC. **Figure S4.** Significantly regulated genes in PMN- and M-MDSC upon poly(I:C)c therapy.
