# Supplementary material for: Immunostimulatory RNA leads to functional reprogramming of myeloid-derived suppressor cells in pancreatic cancer
Source: J Immunother Cancer. 2019 Nov 6;7:288. doi: 10.1186/s40425-019-0778-7 (PMC6836385; doi:10.1186/s40425-019-0778-7)
Supplement: Supplementary file 1 — Additional file 1: Figure S1. Gating strategy for the identification of MDSC populations. Figure S2. Poly(I:C)c reduces macrophage frequency and activates macrophages, cDC, B and NK cells. Figure S3. Poly(I:C)c triggers transcriptional reprogramming of MDSC. Figure S4. Significantly regulated genes in PMN- and M-MDSC upon poly(I:C)c therapy. [file 40425_2019_778_MOESM1_ESM.pdf]

Figure S1

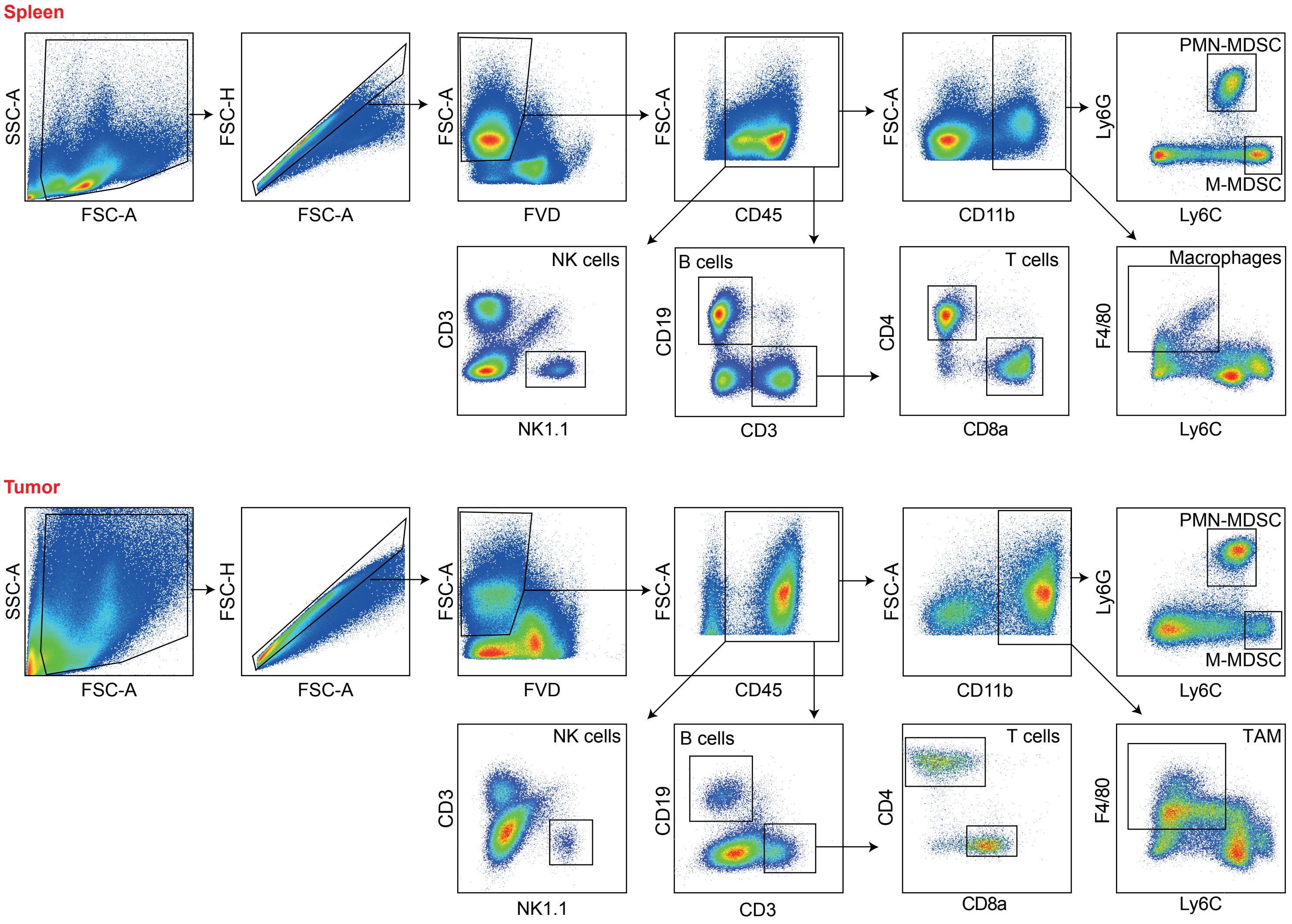
Spleen
SSC-A
FSC-A
FSC-H
FSC-A
FSC-A
FVD
FSC-A
CD45
FSC-A
CD11b
Ly6G
Ly6C
PMN-MDSC
M-MDSC
NK cells
CD3
NK1.1
B cells
CD19
CD3
T cells
CD4
CD8a
Macrophages
F4/80
Ly6C
Tumor
SSC-A
FSC-A
FSC-H
FSC-A
FSC-A
FVD
FSC-A
CD45
FSC-A
CD11b
Ly6G
Ly6C
PMN-MDSC
M-MDSC
NK cells
CD3
NK1.1
B cells
CD19
CD3
T cells
CD4
CD8a
TAM
F4/80
Ly6C

Figure S2

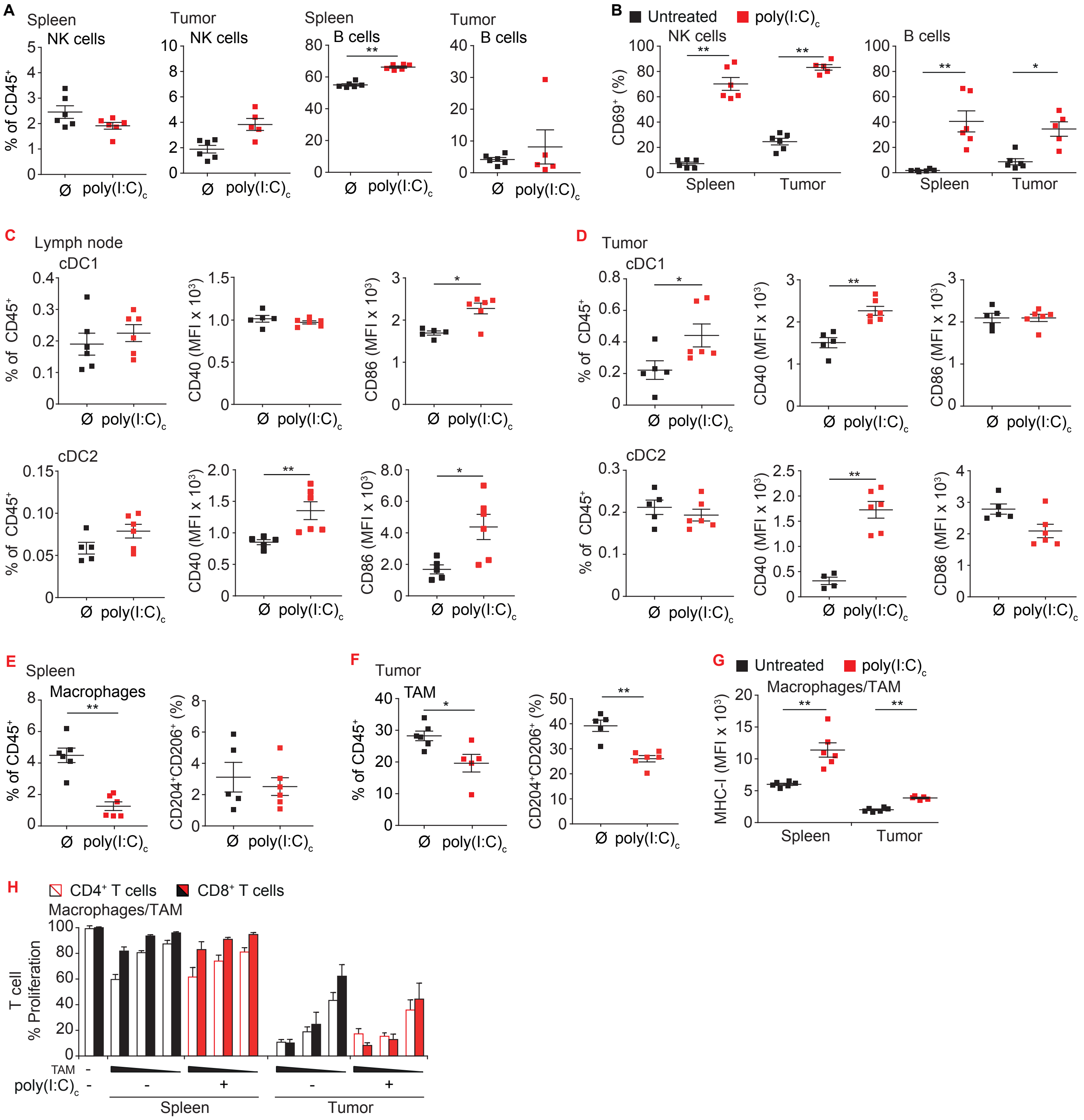

A
Spleen
NK cells
% of CD45⁺
Ø poly(I:C)c
Tumor
NK cells
Spleen
B cells
**
Tumor
B cells
B
Untreated poly(I:C)c
NK cells
CD69⁺ (%)
**
**
Spleen Tumor
B cells
**
*
Spleen Tumor
C
Lymph node
cDC1
% of CD45⁺
CD40 (MFI x 10³)
CD86 (MFI x 10³)
*
cDC2
**
*
D
Tumor
cDC1
*
**
cDC2
**
E
Spleen
Macrophages
**
CD204⁺CD206⁺ (%)
F
Tumor
TAM
*
**
G
Untreated poly(I:C)c
Macrophages/TAM
MHC-I (MFI x 10³)
**
**
Spleen Tumor
H
CD4⁺ T cells CD8⁺ T cells
Macrophages/TAM
T cell % Proliferation
TAM
poly(I:C)c
Spleen
Tumor

Figure S3

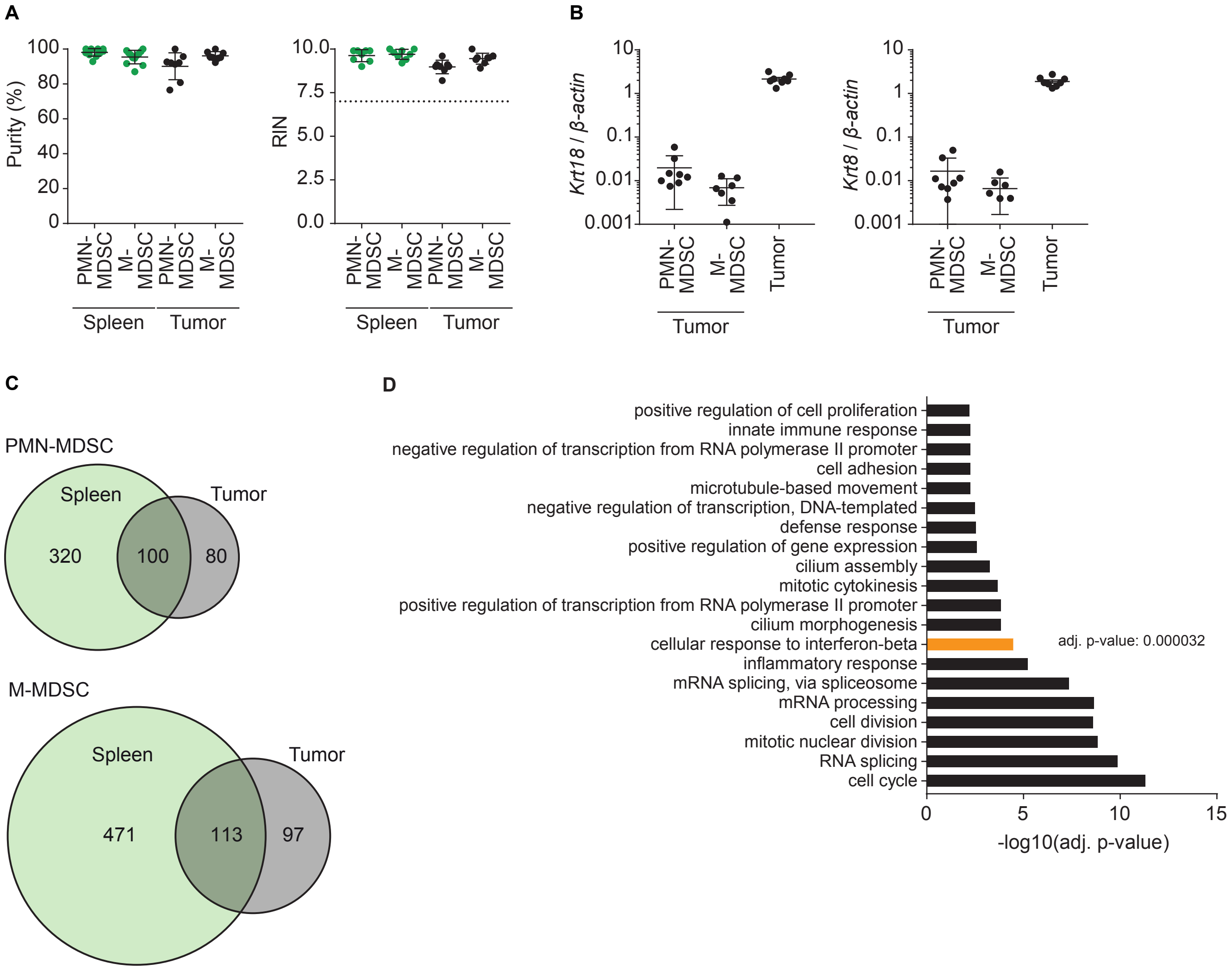
A
Purity (%)
100
80
60
40
20
0
PMN-MDSC
M-MDSC
PMN-MDSC
M-MDSC
Spleen
Tumor
RIN
10.0
7.5
5.0
2.5
0.0
B
Krt18 / β-actin
Krt8 / β-actin
10
1
0.1
0.01
0.001
Tumor
C
PMN-MDSC
Spleen
Tumor
320
100
80
M-MDSC
471
113
97
D
positive regulation of cell proliferation
innate immune response
negative regulation of transcription from RNA polymerase II promoter
cell adhesion
microtubule-based movement
negative regulation of transcription, DNA-templated
defense response
positive regulation of gene expression
cilium assembly
mitotic cytokinesis
positive regulation of transcription from RNA polymerase II promoter
cilium morphogenesis
cellular response to interferon-beta
adj. p-value: 0.000032
inflammatory response
mRNA splicing, via spliceosome
mRNA processing
cell division
mitotic nuclear division
RNA splicing
cell cycle
0
5
10
15
-log10(adj. p-value)

Figure S4

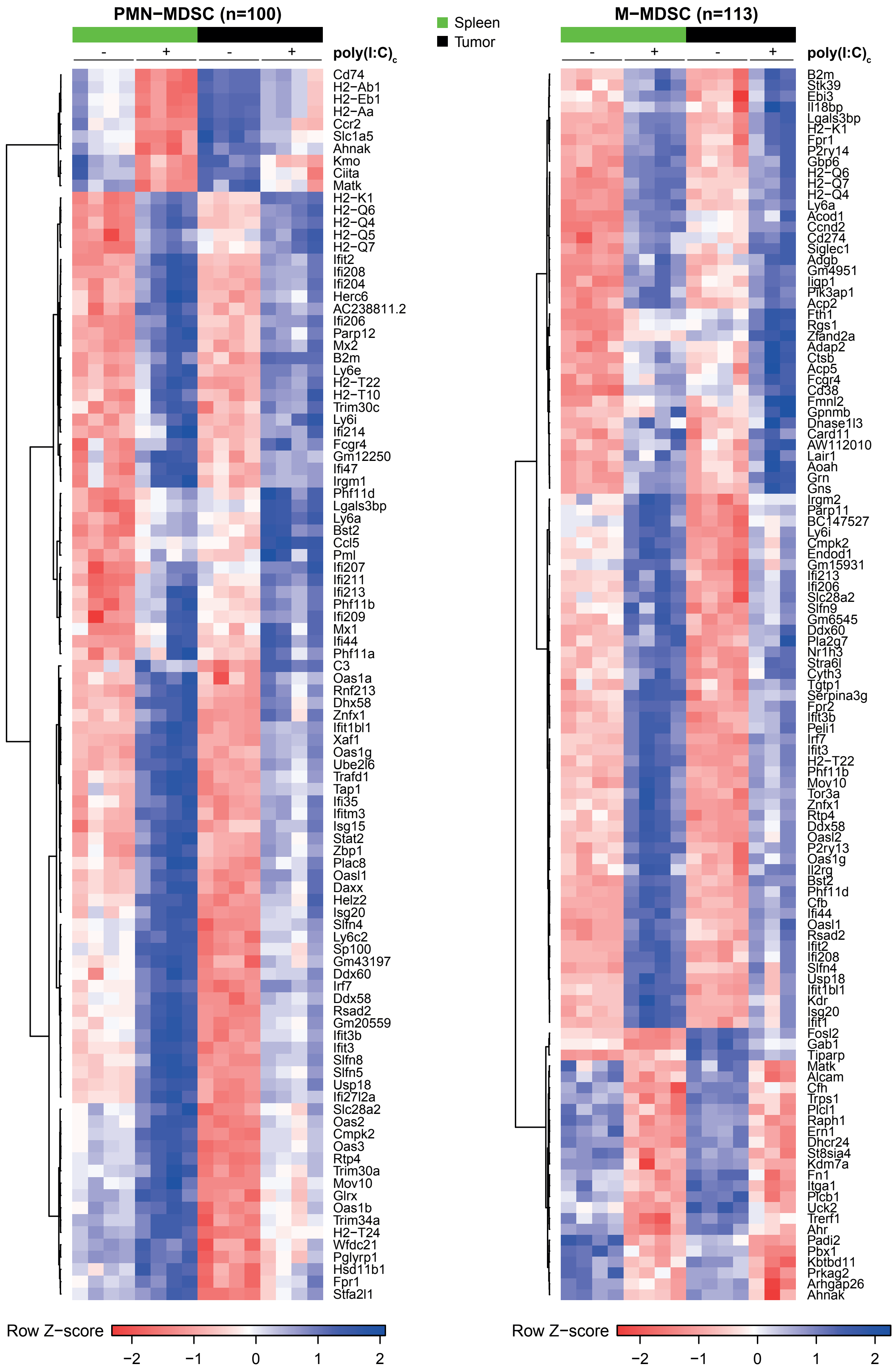

PMN−MDSC (n=100)
M−MDSC (n=113)
Spleen
Tumor
poly(I:C)$_c$
-
+
-
+
Cd74
H2−Ab1
H2−Eb1
H2−Aa
Ccr2
Slc1a5
Ahnak
Kmo
Ciita
Matk
H2−K1
H2−Q6
H2−Q4
H2−Q5
H2−Q7
Ifit2
Ifi208
Ifi204
Herc6
AC238811.2
Ifi206
Parp12
Mx2
B2m
Ly6e
H2−T22
H2−T10
Trim30c
Ly6i
Ifi214
Fcgr4
Gm12250
Ifi47
Irgm1
Phf11d
Lgals3bp
Ly6a
Bst2
Ccl5
Pml
Ifi207
Ifi211
Ifi213
Phf11b
Ifi209
Mx1
Ifi44
Phf11a
C3
Oas1a
Rnf213
Dhx58
Znfx1
Ifit1bl1
Xaf1
Oas1g
Ube2l6
Trafd1
Tap1
Ifi35
Ifitm3
Isg15
Stat2
Zbp1
Plac8
Oasl1
Daxx
Helz2
Isg20
Slfn4
Ly6c2
Sp100
Gm43197
Ddx60
Irf7
Ddx58
Rsad2
Gm20559
Ifit3b
Ifit3
Slfn8
Slfn5
Usp18
Ifi27l2a
Slc28a2
Oas2
Cmpk2
Oas3
Rtp4
Trim30a
Mov10
Glrx
Oas1b
Trim34a
H2−T24
Wfdc21
Pglyrp1
Hsd11b1
Fpr1
Stfa2l1
B2m
Stk39
Ebi3
Il18bp
Lgals3bp
H2−K1
Fpr1
P2ry14
Gbp6
H2−Q6
H2−Q7
H2−Q4
Ly6a
Acod1
Ccnd2
Cd274
Siglec1
Adgb
Gm4951
Iigp1
Pik3ap1
Acp2
Fth1
Rgs1
Zfand2a
Adap2
Ctsb
Acp5
Fcgr4
Cd38
Fmnl2
Gpnmb
Dnase1l3
Card11
AW112010
Lair1
Aoah
Grn
Gns
Irgm2
Parp11
BC147527
Ly6i
Cmpk2
Endod1
Gm15931
Ifi213
Ifi206
Slc28a2
Slfn9
Gm6545
Ddx60
Pla2g7
Nr1h3
Stra6l
Cyth3
Tgtp1
Serpina3g
Fpr2
Ifit3b
Peli1
Irf7
Ifit3
H2−T22
Phf11b
Mov10
Tor3a
Znfx1
Rtp4
Ddx58
Oasl2
P2ry13
Oas1g
Il2rg
Bst2
Phf11d
Cfb
Ifi44
Oasl1
Rsad2
Ifit2
Ifi208
Slfn4
Usp18
Ifit1bl1
Kdr
Isg20
Ifit1
Fosl2
Gab1
Tiparp
Matk
Alcam
Cfh
Trps1
Plcl1
Raph1
Ern1
Dhcr24
St8sia4
Kdm7a
Fn1
Itga1
Plcb1
Uck2
Trerf1
Ahr
Padi2
Pbx1
Kbtbd11
Prkag2
Arhgap26
Ahnak
Row Z−score
−2
−1
0
1
2
Row Z−score
−2
−1
0
1
2
