# Supplementary figures and images for: Correction to: Immunostimulatory RNA leads to functional reprogramming of myeloid-derived suppressor cells in pancreatic cancer
Source: J Immunother Cancer. 2019 Dec 16;7:349. doi: 10.1186/s40425-019-0830-7 (PMC6916026; doi:10.1186/s40425-019-0830-7)

Figure S1

Spleen

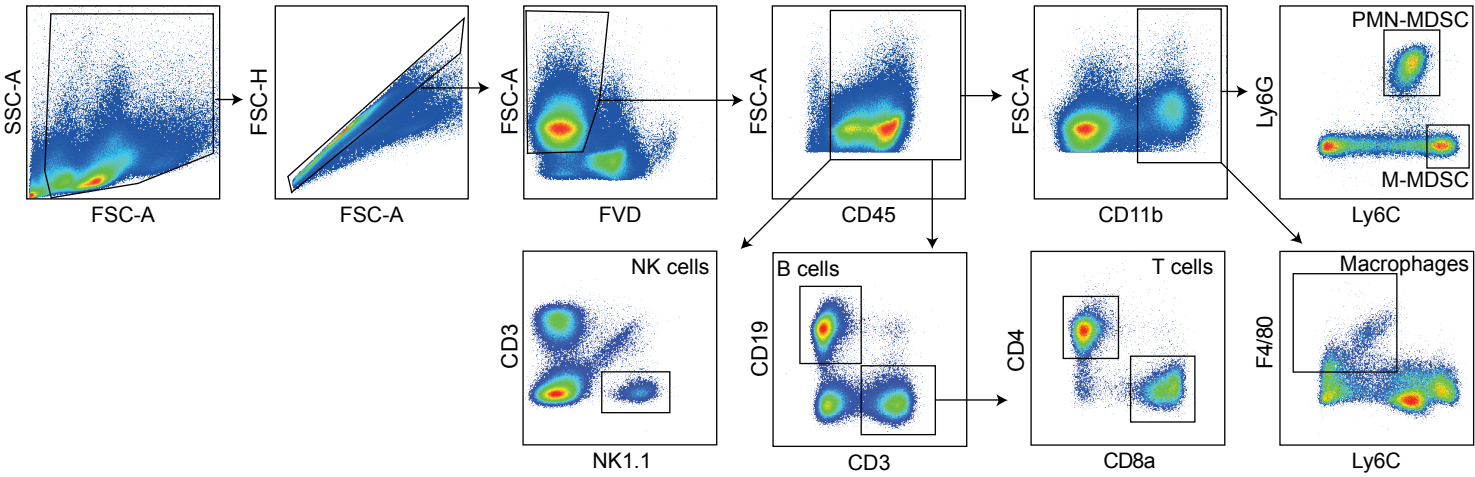

Tumor

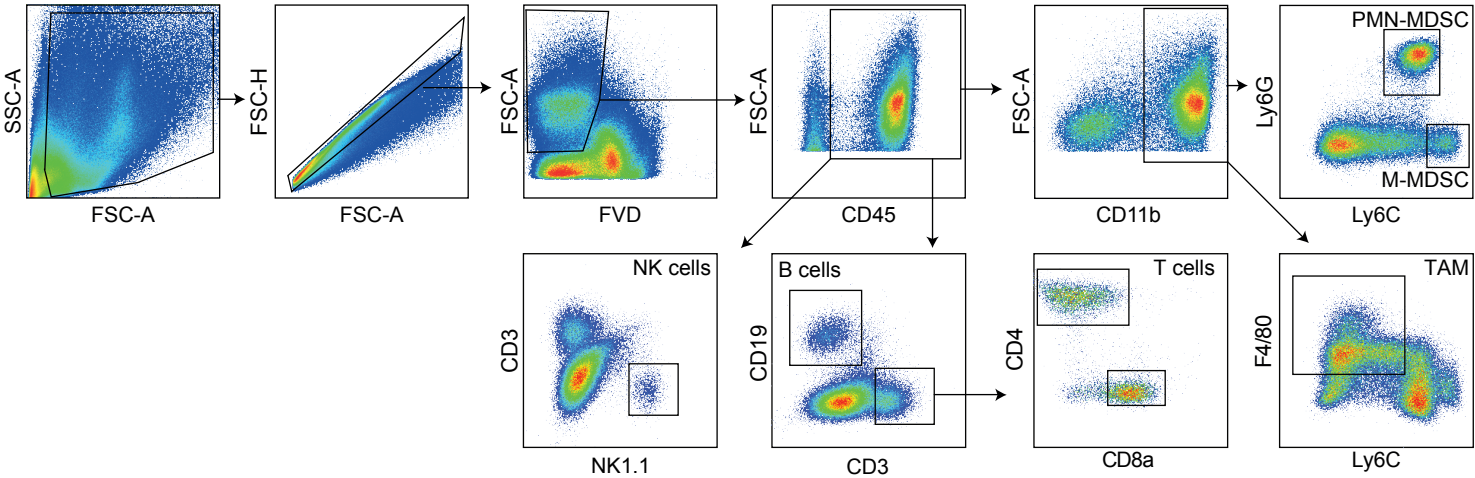

**Figure S2**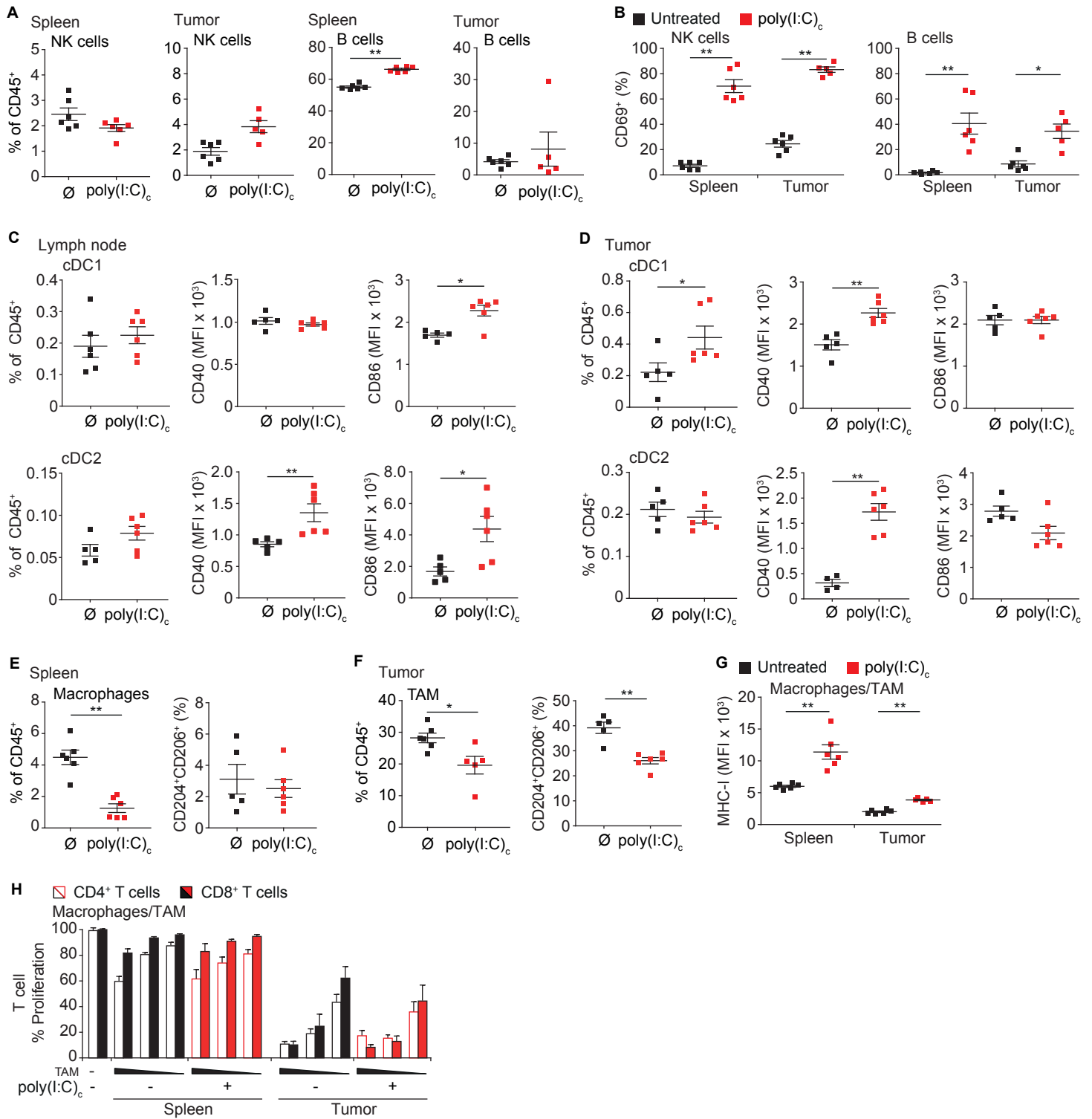

Figure S3

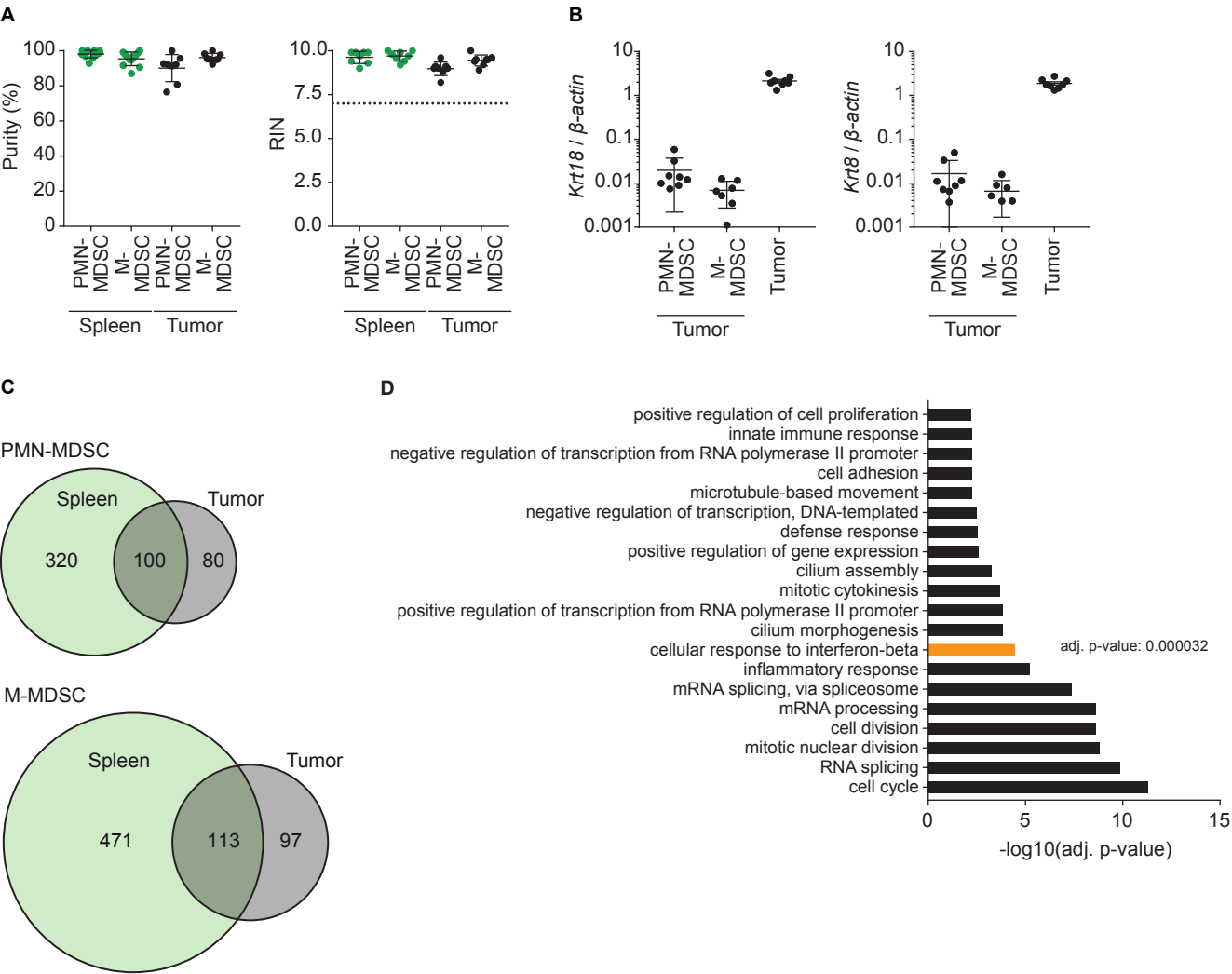

Figure S4

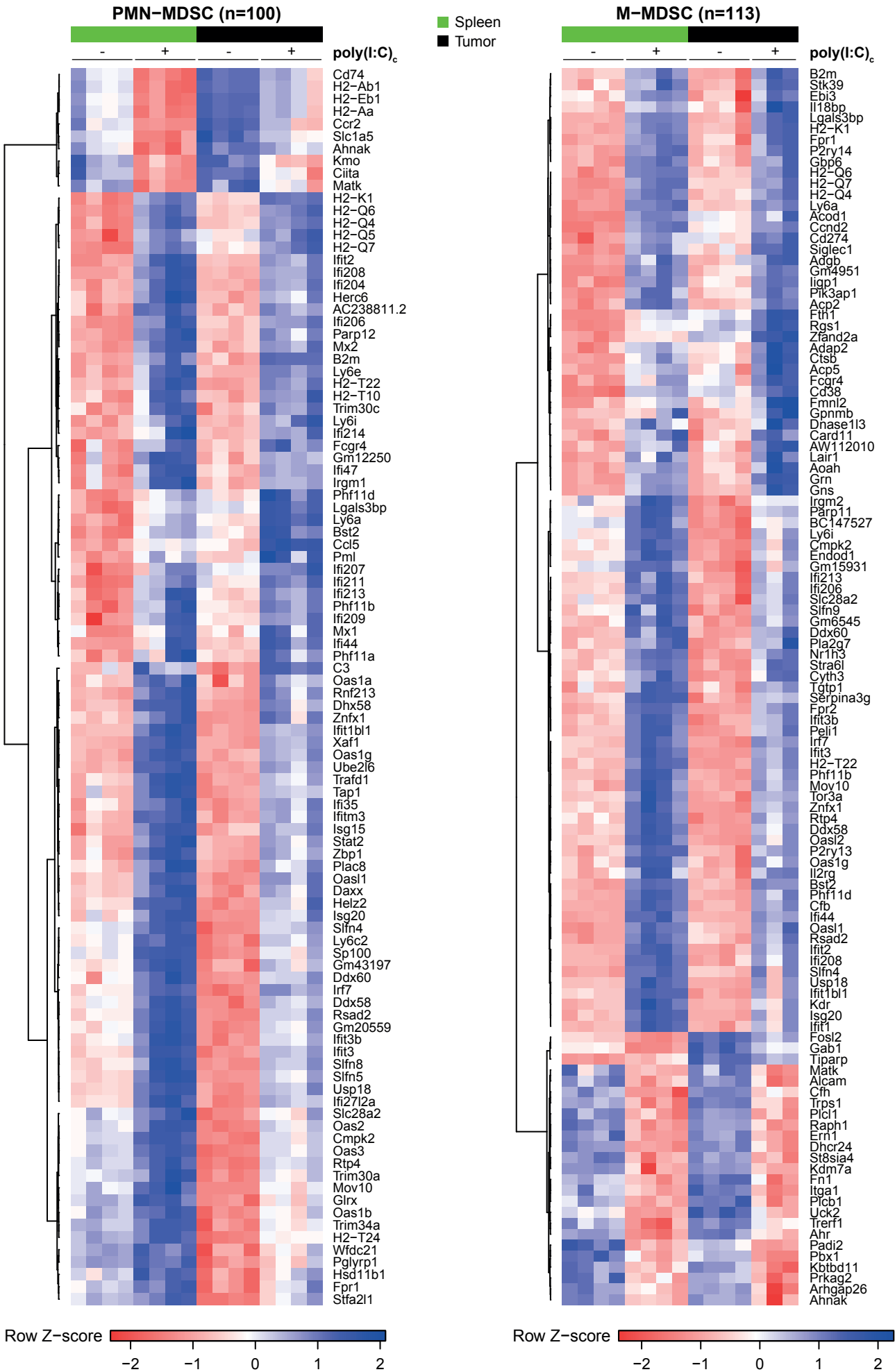

Supplement: Supplementary file 1 — Additional file 1: Figure S1.. Gating strategy for the identification of MDSC populations. Figure S2. Poly(I:C)c reduces macrophage frequency and activates macrophages, cDC, B and NK cells. Figure S3. Poly(I:C)c triggers transcriptional reprogramming of MDSC. Figure S4. Significantly regulated genes in PMN- and M-MDSC upon poly(I:C)c therapy. [file 40425_2019_830_MOESM1_ESM.pdf]
